# Novel partiti-like viruses are conditional mutualistic symbionts in their normal lepidopteran host, African armyworm, but parasitic in a novel host, Fall armyworm

**DOI:** 10.1371/journal.ppat.1008467

**Published:** 2020-06-22

**Authors:** Pengjun Xu, Liyu Yang, Xianming Yang, Tong Li, Robert I. Graham, Kongming Wu, Kenneth Wilson

**Affiliations:** 1 Tobacco Research Institute, Chinese Academy of Agricultural Sciences, Qingdao, China; 2 Lancaster Environment Centre, Lancaster University, Lancaster, United Kingdom; 3 State Key Laboratory for Biology of Plant Diseases and Insect Pests, Institute of Plant Protection, Chinese Academy of Agricultural Sciences, Beijing, China; 4 Institute of Plant Protection, Henan Academy of Agricultural Sciences, Zhengzhou, China; 5 Department of Animal and Agriculture, Hartpury University, Gloucester, United Kingdom; University of Edinburgh, UNITED KINGDOM

## Abstract

Recent advances in next generation sequencing (NGS) (e.g. metagenomic and transcriptomic sequencing) have facilitated the discovery of a large number of new insect viruses, but the characterization of these viruses is still in its infancy. Here, we report the discovery, using RNA-seq, of three new partiti-like viruses from African armyworm, *Spodoptera exempta* (Lepidoptera: Noctuidae), which are all vertically-transmitted transovarially from mother to offspring with high efficiency. Experimental studies show that the viruses reduce their host’s growth rate and reproduction, but enhance their resistance to a nucleopolyhedrovirus (NPV). Via microinjection, these partiti-like viruses were transinfected into a novel host, a newly-invasive crop pest in sub-Saharan Africa (SSA), the Fall armyworm, *S*. *frugiperda*. This revealed that in this new host, these viruses appear to be deleterious without any detectable benefit; reducing their new host’s reproductive rate and increasing their susceptibility to NPV. Thus, the partiti-like viruses appear to be conditional mutualistic symbionts in their normal host, *S*. *exempta*, but parasitic in the novel host, *S*. *frugiperda*. Transcriptome analysis of *S*. *exempta* and *S*. *frugiperda* infected, or not, with the partiti-like viruses indicates that the viruses may regulate pathways related to immunity and reproduction. These findings suggest a possible pest management strategy via the artificial host-shift of novel viruses discovered by NGS.

## Author summary

Modern molecular approaches allow the discovery of new viruses that do not present obvious symptoms of disease in their hosts. Using these methods, we discovered three new partiti-like viruses in a major crop pest, the African armyworm, *Spodoptera exempta*, which are naturally transmitted vertically from adult females to their offspring in her eggs. We discovered that these viruses decrease their host’s growth rate and reproduction, but they may also help their insect host by protecting them from other lethal viruses, specifically the baculoviruses that are often used as biological pesticides. We transferred these partiti-like viruses into a novel host, the Fall armyworm, *S*. *frugiperda*, by microinjection and found that they also decrease *S*. *frugiperda*’s growth rate and reproduction. Interestingly, however, it appears that the partiti-like viruses make *S*. *frugiperda* more susceptible to baculovirus, potentially making it more vulnerable to control using biopesticides. Our results suggest a possible novel pest management strategy via artificial host-shift of viruses discovered using molecular approaches and offers new insights into host-virus-virus interactions.

## Introduction

The development of next generation sequencing (NGS) technologies has facilitated the discovery and analysis of novel pathogens with or without overt symptoms, e.g. thousands of viruses have been reported in vertebrate and invertebrate species using metagenomic and transcriptomic sequencing technologies [1,2,3,4,5]. Insect pathogens (including bacteria, fungi and viruses) that naturally kill their hosts can be harnessed as potential biopesticides [6,7,8]. The identification of potential biocontrol agents of insects has largely relied on isolating overtly pathogenic microorganisms from cadavers. Symbionts (e.g. bacteria and viruses) form diverse relationships with their insect hosts: *parasitic*, by killing them or reducing their fertility [9,10]; *mutualistic*, by increasing their host’s fitness [11,12,13,14]; and *conditional mutualistic*, by being either beneficial or harmful to their host depending on context, e.g. host genotype [15]. Moreover, co-infection of symbionts may alter the levels of resistance of their hosts to other pathogens, including those used in biopesticides (e.g. baculoviruses) [9,12]. Consequently, the identification of novel symbionts using NGS raises a number of questions. First, can we find new pathogens for pest management by determining the interactions between these symbionts and their hosts? Second, how do these newly-discovered symbionts interact with known pathogens, including biopesticides (e.g. baculoviruses)? And third, how do these symbionts behave in novel hosts to which they have not co-evolved? This latter question is particularly pertinent to newly-introduced invasive host species, which may have fewer parasites in the new range than in their native range [16], but may also become exposed to novel parasites from other host species in their new environment [17,18,19].

The African armyworm, *S*. *exempta*, is an important migratory crop pest of cereals in sub-Saharan Africa (SSA), including maize, millet, rice, wheat, etc. [20]. It plays host to an endemic baculovirus, *Spodoptera exempta* nucleopolyhedrovirus (SpexNPV) that has been developed as a potential biopesticide [9,21,22], providing a useful model system for addressing the questions posed above. In this study, we used NGS technologies and bioinformatic approaches to search for novel viruses in *S*. *exempta*, revealing three new partiti-like viruses. By microinjection, the partiti-like viruses could infect both *S*. *exempta* and *S*. *frugiperda* (the Fall armyworm), a polyphagous pest originating from tropical/subtropical regions of the Americas, that has recently spread rapidly to other parts of the world, including SSA where its new range overlaps with that of *S*. *exempta* [23,24,25,26,27]. The Fall armyworm also has an endemic baculovirus that has been used as an effective biopesticide in the Americas [28,29,30]. Bioassays reveal for the first time that both *S*. *exempta* and *S*. *frugiperda* infected with these newly-discovered partiti-like viruses developed more slowly and had lower fertility. However, the two host species differed in the effect of the partiti-like viruses on susceptibility to baculovirus, with it decreasing susceptibility in *S*. *exempta* but increasing it in *S*. *frugiperda*. The transcriptional profiles of infected and non-infected individuals were consistent with the phenotypes of these two species having different gene expression profiles in response to infection by partiti-like viruses. These results suggest that the partiti-like viruses are conditional mutualistic symbionts in *S*. *exempta*, protecting it when challenged with baculovirus, but are parasitic in its novel host, *S*. *frugiperda*.

## Material and methods

### Insect culture and detection of viruses by RNA-seq

The colonies of *S*. *exempta* were established with individuals collected in South Africa in 2014 and Tanzania in 2017; those of *S*. *frugiperda* and *S*. *littoralis* were established with individuals collected in Zambia in 2017 and Egypt in 2011, respectively; and the *H*. *armigera* colony was established with individuals from Andermatt Biocontrol AG (Switzerland) in 2018. All larvae were reared in the lab using standard semi-artificial wheatgerm-based diet at 26°C with a 14:10, light:dark photoperiod, as described previously [9]. Adult moths were provided with 5% sugar water. Eggs and pupae were not routinely treated with sodium hypochlorite, but see below.

To detect viruses with poly(A), we used the whole bodies of *S*. *exempta*, including first instar larvae (n = 50), fifth instar larvae (n = 20), pupae (n = 20) and adults (n = 20), using individuals from the two colonies equally. The transcriptome was sequenced with two paired-end and 150-nt read length on the channels of an Illumina HiSeq instrument (Majorbio, Shanghai, China). Briefly, for first instar larvae, 50 individuals were ground up under liquid nitrogen and put into a 1.5 ml tube with 1 ml TRIzol *(*Invitrogen, Grand Island, USA*)*. For fifth instar larvae, and adult males and females, individuals were ground up separately and put into a 1.5 ml tube with 1 ml TRIzol. After vortex and centrifugation for 5 min at 12000×g, 50 μl from first instar larvae and 20 μl per individual from other samples were combined together for extracting total RNA. The cDNA library was prepared with TruSeq RNA sample preparation Kit from Illumina (San Diego, CA) according to the manufacturers instructions. Soon after, mRNA was isolated from 5 μg total RNA using Oligo (dT) magnetic beads and then was fragmented (about 200bp) in fragmentation buffer. These short fragments were used as templates for double-stranded cDNA synthesis using a SuperScript double-stranded cDNA synthesis kit (Invitrogen) with random hexamer-primers. The cDNA was then subjected to end-repair and single nucleotide adenine addition. Suitable fragments (200–300 bp) judged by agarose gel electrophoresis were enriched with PCR amplification to prepare the sequencing library and the library was sequenced on the with Illumina HiSeq platform for about 6 gigabase in-depth. To detect whether the *S*. *frugiperda* colony was infected by viruses, samples with the same number and method used in *S*. *exempta* were collected and sequenced accordingly. The Trinity (v2.0.6) software was used to assemble the clean reads and unigenes were generated using contigs longer than 200 bp with default parameters (kmer = 5) [31]. Unigenes were annotated with protein databases. Briefly, unigenes were searched against the NCBI protein non-redundant (NR), SwissProt and clusters of eukaryotic Orthologous Groups (KOG) databases using DIAMOND (v0.8.37) (e-value < 1E-5) [32], the Kyoto Encyclopedia of Genes and Genomes (KEGG) database using KAAS (r140224) (default) [33], the Gene Ontology (GO) database using Blast2GO (v2.5.0) (default) [34], the Interpro database using InterProScan5 (V5.11–51.0) (default) [35], and the Pfam database using HMMER 3 (v3.1b2) (default) [36]. The RNA-Seq data were submitted to the NCBI Sequence Read Archive (SRA) database (S1 Table).

### Partiti-like viruses detection, preparation and quantification

Individuals were ground up under liquid nitrogen and RNA was extracted to synthesize cDNA templates for virus detection: 2 μg total RNA were used to synthesize cDNA template of 25 μl per sample and 2 μl cDNA was used per PCR reaction. All of the primers used in this study are shown in S2 Table. To detect the three viruses we found in *S*. *exempta* by transcriptome, specific primers amplifying 936 bp, 944 bp and 867 bp fragments for *S*. *exempta* virus 1 (SEIV1), *S*. *exempta* virus 2 (SEIV2) and *S*. *exempta* virus 3 (SEIV3) respectively were designed according to the genomic sequences of viruses from the RNA-seq data. The PCR program was as follows: 30 s at 94 ^o^C, 30 s at 55 ^o^C, and 45 s at 72 ^o^C for 40 cycles. The fragments were cloned into the pEASY-T Cloning Vector (TransGen, Beijing, China). These plasmids were subsequently used for the quantification standard curve assay. We also designed primers to detect and exclude the infection of *S*. *exempta* plant-fungal virus-like virus (SEPV) in this study. Specific primers amplifying 730 bp fragment for SEPV were designed according to the genomic sequences from the RNA-seq data. The PCR program was as follows: 30 s at 94 ^o^C, 30 s at 50 ^o^C, and 45 s at 72 ^o^C for 40 cycles.

For amplifying the 3’ ends of the three viruses, cDNA templates were synthesized using total RNA extracted from partiti-like viruses-positive samples with primer 3CDS and the PCRs were performed with specific primers and 3UPM as follows: 30 s at 94 ^o^C, 3 min at 72 ^o^C for 5 cycles; 30 s at 94 ^o^C, 30 s at 70 ^o^C, and 3 min at 72 ^o^C for 5 cycles; 30 s at 94 ^o^C, 30 s at 68 ^o^C, and 3 min at 72 ^o^C for 30 cycles. For amplifying the 5’ ends of the three viruses, cDNA templates were synthesized with primer Oligo(dT) and then, were added poly(A) at the 5’ ends using terminal deoxyribonucleotidyl transferase and dATP (Takara, Japan). The PCRs were performed with specific primers and 5UPM under the same condition as amplification of 3’ ends. The PCR products were cloned into the pEASY-T cloning vector and were sequenced (TransGen).

Partiti-like viruses were isolated from positive individuals lacking SEPV. Briefly, individuals were ground up under liquid nitrogen and part of them was used for RNA extraction, and PCR undertaken to detect the presence of the viruses. Subsequently, the remainder tissues of positive individuals were used to prepare a filtered liquid containing the viruses. Briefly, about 400 mg of tissues were transferred to 1 ml PBS buffer (0.01M, pH 7.4). The homogenate was centrifuged at 6500×g for 15 min at 4°C, and the liquid supernatant subsequently filtered with *Sartorius Minisart 0*.*2* μ*m PES (*Invitrogen). All the samples were stored at -20°C.

Using β-actin and GAPDH as references genes, we chose two viruses (SEIV1 and SEIV2) for quantifying the relative expression level of the viruses in *S*. *exempta* with 2×Premix Ex Taq (Takara, Japan) and 7500 Fast Real-time PCR System (Applied Biosystems). qPCR was carried out with the TaqMan method in 20 μl reaction agent comprised of 1 μl of template DNA, 2×Premix Ex Taq (Takara), 0.2 μM of each primer and 0.4 μM probe. Thermal cycling conditions were: 45 cycles of 95°C for 15 s, 60°C for 34 s. Each sample was replicated three times and there were more than three biological replicates for each point. The absolute quantification qPCR methodology was used to quantify the copy numbers of the two viruses using the same method as used previously for HaDV2 [12].

### Phylogenetic analysis

The amino acid sequences of RNA-dependent RNA polymerase (RdRp) conserved domains of the three partiti-like viruses, as well as other viruses from the family *Partitiviridae* and the Partiti-Picobirna group [1,5,37], were aligned with MUSCLE method in MEGA 7.0 [38]. The poorly aligned regions were further removed with trimAI [39]. A phylogenetic tree was constructed using maximum likelihood methods in IQ-TREE 1.6.6 [40]. The best fit substitution model was selected with Bayesian information criterion in ModelFinder [41]. The ultrafast bootstrap method with 5000 replicates was used to assess the support for each node [42].

### Partiti-like viruses transmission and host tissue distribution

Firstly, we constructed strains of *S*. *exempta*, *S*. *frugiperda*, *S*. *littoralis*, and *H*. *armigera* that were assumed free of the partiti-like viruses, SEPV, NPV and *Wolbachia* from single uninfected breeding pairs without eggs or pupae surface-sterilization. The partiti-like viruses were detected as described above and the infection statuses of SEPV, NPV and *Wolbachia* were confirmed with specific primers and PCR program: 30 s at 94 ^o^C, 30 s at 50/55 ^o^C, and 30 s at 72 ^o^C for 40 cycles; and assumed to be free of these microbes if no PCR amplification was detected at 10ng host DNA. Two methods (peroral infection and microinjection) were used for detection of horizontal transmission efficiency of the partiti-like viruses. Briefly, for peroral infection, about 100 μl of filtered liquid from partiti-like viruses infected larvae was put on the surface of a 2.8 cm diameter pot containing a semi-synthetic wheatgerm based diet. After drying, ten virus-negative neonates were placed on the diet for 48 h. They were then transferred to a 25-well plate (one individual per 2 cm diameter well) until the 4^th^ larval instar; larvae were then individually reared in 2.8 cm diameter plastic diet pots until eclosion. For microinjection, each newly moulted 5^th^ instar larva was injected with 10 μl filtered liquid containing the three partiti-like viruses with a Hamilton Microliter (705N) syringe and Harvard Pump 11 Elite. Then, the larvae were reared as described above until eclosion and the moths were used to detect the viruses with PCR and specific primers. Individuals from the partiti-like viruses positive strains constructed with microinjection were used to determine the vertical transmission mode in *S*. *exempta*. F+/M-, F-/M+, F+/M+ and F-/M- pairs were crossed and RNA from offspring adults was used to probe for the viruses.

To examine the partiti-like viruses infection in different stages and body tissues, RNA was extracted from whole body at different stages (1^st^-day individuals, n = 4) and body parts (1^st^-day larvae at 5^th^ instar stage (n ≥ 3), 1^st^-day females and 1^st^-day males (n = 3) of infected individuals and the viruses were quantified by qPCR with β-actin and GAPDH as reference genes.

### Host range of the partiti-like viruses

Using the same microinjection method as previously described, we chose three species of Lepidoptera (*S*. *frugiperda*, *S*. *littoralis*, and *H*. *armigera*) to determine the host range of the partiti-like viruses. Firstly, we constructed colonies with offspring from a single uninfected breeding pair of the three species as well as excluding the possibility of SEPV, NPV and *Wolbachia* infection, as described above. After microinjection, the moths were screened for the presence of the partiti-like viruses. The vertical transmission efficiency was also quantified using the same method as for *S*. *exempta*.

### Quantification of the partiti-like viruses in eggs

To further establish the role of vertical transmission in the life-cycle of the partiti-like viruses, we quantified the infections in *S*. *exempta* and *S*. *frugiperda* eggs, primarily to distinguish between *transovarial* and *transovum* infection routes. Eggs from positive strain breeding pairs, in which both of females and males were infected with the partiti-like viruses, were submerged in 5% sodium hypochlorite for 10 minutes. They were then filtered through a damp cloth, thoroughly rinsed, and allowed to dry. Four groups of hypochlorite-treated eggs from *S*. *exempta* and *S*. *frugiperda* (n = 50 eggs per group) were tested against non-treated eggs (control) the infections tested by qPCR.

### Quantifying the impact of partiti-like viruses on host development and fecundity

With microinjection, we successfully constructed the partiti-like viruses-positive strains using negative individuals from single pairs in *S*. *exempta* and *S*. *frugiperda*. To test the impact of the partiti-like viruses infections on the life history parameters of *S*. *exempta* and *S*. *frugiperda*, including development, fecundity, larval mortality, pupation rate and eclosion rate, twenty neonate larvae from these partiti-like viruses positive or negative strains were placed in each diet pot for three days. They were then transferred to a 25-well plate until the 4^th^ larval instar, when they were then individually reared in plastic pots until eclosion. The status of individuals was checked every day at 10:00 am. The weight of pupae on the 1^st^ day was recorded. Individuals dying within 24 hours of the experimental set up were considered handling deaths, and excluded from the analysis.

In addition, newly eclosed adults from both the partiti-like viruses-negative and -positive strains were mated (F+/M+ and F-/M-) and used to determine longevity and fertility. A single pair of adults was put in each plastic cup (diameter = 11 cm; height = 6 cm). The experimental replicates were no less than 20 for each strain respectively.

### Baculovirus bioassays

To assess the effect of partiti-like viruses on susceptibility to NPV in their common hosts, we quantified the interaction between the partiti-like viruses and NPV in the two *Spodoptera* species via a series of laboratory bioassays using *Spodoptera exempta* nucleopolyhedrovirus (SpexNPV) and *Spodoptera frugiperda* multiple nucleopolyhedrovirus (SfMNPV) for *S*. *exempta* and *S*. *frugiperda*, respectively. As previously described, 20 neonate larvae from these partiti-like viruses positive or negative strains were placed in each diet pot for three days. They were then transferred to a 25-well plate and maintained on diet. On the first day of the 4^th^ instar, *S*. *exempta* and *S*. *frugiperda* larvae were orally challenged with diet plugs and one of four doses of SpexNPV (no less than 60 larvae per treatment at: 0 (control), 1×10^3^, 5×10^3^, 2.5×10^4^, and 1×10^5^ occlusion bodies (OBs)/larva) or SfMNPV (no less than 80 larvae per treatment at: 0 (control), 8×10^3^, 4×10^4^, 2×10^5^, and 1×10^6^ OBs/larva). The diet plugs are no bigger than 2×2 mm and carefully pipette 1 μl of treatment virus solution onto per diet plug. Only larvae that ingested all the NPV within a 24 h period were used for the bioassays. Larvae were subsequently monitored daily for NPV mortality until pupation, and all NPV-killed cadavers stored at -20°C. PCR with specific primers was used to test for NPV in dead larvae with non-obvious symptoms.

To assess NPV infection levels in the partiti-like viruses-positive and -negative individuals in the two species, we performed a separate bioassay with the same method of diet plug and 2.5×10^4^ OBs/larva in *S*. *exempta* and 2×10^5^ OBs/larva in *S*. *frugiperda*. Only larvae that ingested all of the NPV within a 24 h period were used and collected samples at 72 h after ingesting all the diet. To perform the absolute quantification qPCR methodology for quantifying NPV copy number, firstly we designed primers according to the open reading frame encoding polyhedrin and amplified fragments for constructing plasmids to generate standard curve (706 bp for SpexNPV and 756 bp for SfMNPV) as described above. The PCR program was as follows: 30 s at 94 ^o^C, 30 s at 55 ^o^C, and 30 s at 72 ^o^C for 40 cycles.

### Analyzing effects of the partiti-like viruses on its hosts by transcriptome

To determine the effect of these partiti-like viruses on the two *Spodoptera* species at a transcriptomic level, we collected samples of the partiti-like viruses-negative and -positive individuals from single pairs of *S*. *exempta* and *S*. *frugiperda* and performed RNA-seq, using first-day fifth instar larvae, pupae, males and females. There were three groups and nine individuals for each group per stage, except for virus-negative pupae in *S*. *exempta* and males in *S*. *frugiperda*, which had two groups (S1 Table). The cDNA libraries were constructed, sequenced and the data were assembled and annotated as described above. For the gene expression analysis, the number of expressed tags was calculated and then normalized to transcripts per million tags (TPM) using RSEM software packages [43]. Then the R package *edgeR* [44] was used to determine the significantly differentially-expressed unigenes at different comparisons with threshold ‘fold change ≥ 1.5 and P<0.05. The hierarchical clustering method was applied to analyze the expression pattern of significantly differentially expressed unigenes in different samples. The statistical significance of the functional GO enrichment was evaluated using the Fishers exact test with python package *Goatools* (p-values were corrected by the Benjamini-Hochberg, false discovery rate (FDR) < 0.05) [45]. Significantly enriched KEGG pathways were also identified using the Fishers exact test (p-value<0.05). Using β-actin and GAPDH as reference genes, qPCR with Sybgreen method was performed to confirm the results of RNA-seq in 20 μl reaction agent comprised of 1 μl of template DNA, 2×Premix Ex Taq (Takara), 0.2 μM of each primer, using a 7500 Fast Real-time PCR System (Applied Biosystems). Thermal cycling conditions were: 45 cycles of 95°C for 3 s, 60°C for 30 s. The samples of each group were biologically replicated three times.

### Statistics

Statistical analyses were conducted using Graphpad InStat 3 and R v3.0.1 [46]. Student’s t-test or ANOVA with Tukey LSD tests were used to determine the level of significance in the relative levels of the partiti-like virus. Larval/pupal mortality, pupation and eclosion rates, and NPV bioassay data, were analyzed using generalized linear models (GLMs) with binomial errors.

## Results

### Identification of viruses

**Fig 1:**
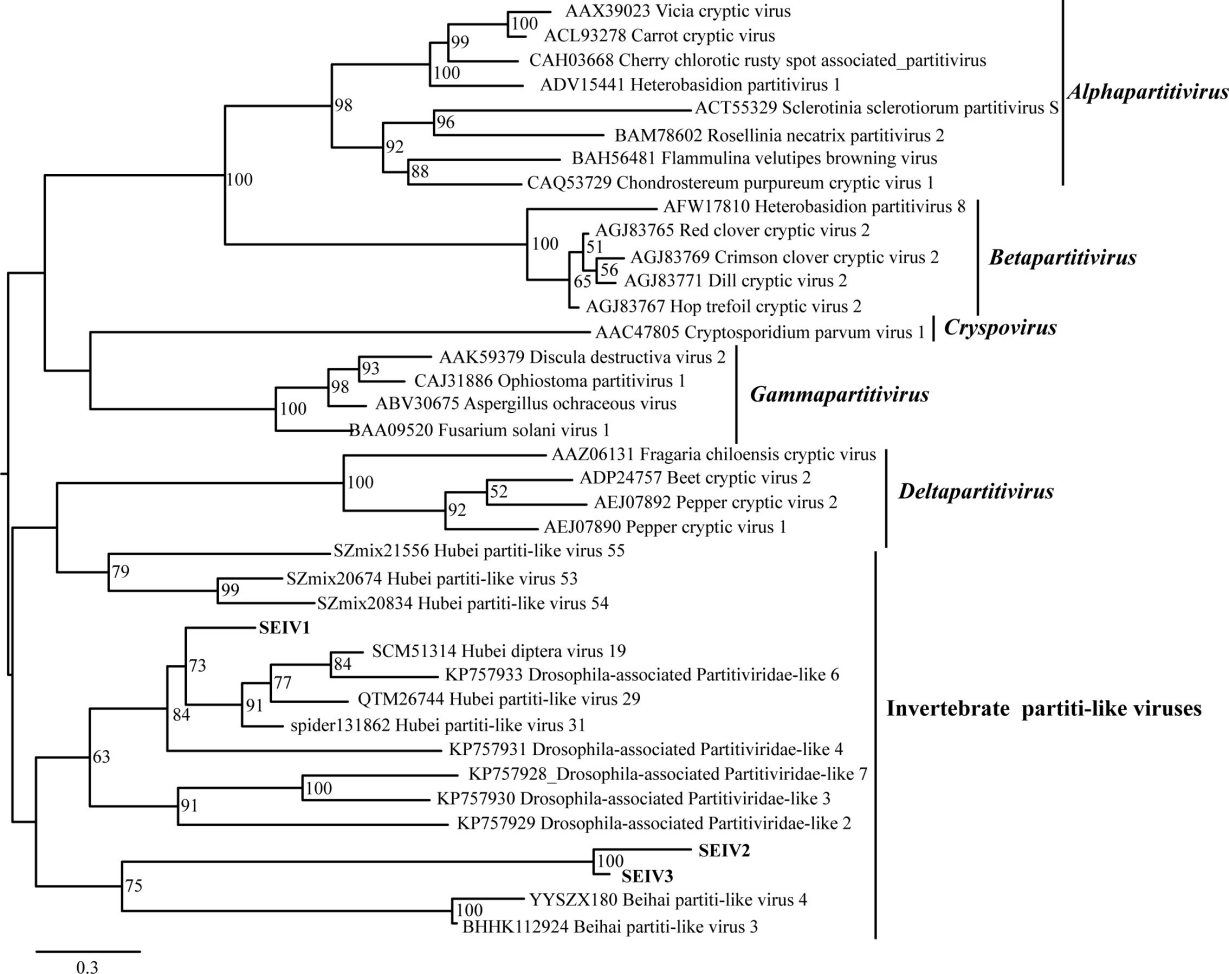
Phylogenetic analysis of partiti-like viruses found in *Spodoptera exempta*. Maximum likelihood tree constructed with IQ-TREE 1.6.6 using amino acid sequences of the conserved RdRp domain of SEIV1, SEIV2, SEIV3, thirteen other partiti-like viruses from invertebrates, and 22 members from the family Partitiviridae. *Alphapartitivirus*, *Betapartitivirus*, *Gammapartitivirus*, *Deltapartitivirus* and *Cryspovirus* are the five genera within the family *Partitiviridae*, the members of which were reported to infect plant, fungi and protists. The bootstrap values (5000 pseudoreplicates) > 50% are indicated on the nodes.

Using RNA-seq (S1 Table), we identified three new partiti-like viruses in *S*. *exempta* (SEIV1, SEIV2 and SEIV3) and one virus showing high identity with viruses from fungal plant pathogens (SEPV); here we focus just on the three partiti-like viruses. We designed primers to detect these viruses in samples from lab-reared and field-collected *S*. *exempta* and only found the three partiti-like viruses-positive individuals from field populations, which were co-infected by all three partiti-like viruses (S2 Table; S1A Fig; S1 Text). We then amplified the complete coding sequence (CDS) containing RdRp conserved domains of the three partiti-like viruses with rapid amplification of cDNA ends method (RACE) (S2 Fig; S2 Table; S1 Text). The substitution model of LG with unequal base frequencies (+F), a proportion of invariable sites (+I), and 4 rate categories of gamma among sites (+G4) was selected for the alignment [47]. The phylogenetic trees, generated using the maximum likelihood method with the support for each node being assessed with the ultrafast bootstrap method with 5000 replicates, indicated that SEIV1, SEIV2 and SEIV3 all clustered with a group of newly-reported partiti-like viruses of invertebrates (Fig 1, S2 Text) [1,37]. SEIV2 and SEIV3 clustered together and the amino acid sequences of the peptides showed high identity with each other (77%), suggesting they are closely related species, so we focused on SEIV1 and SEIV2 to determine the virus’ transmission and host ranges.

### Transmission mode and host-tissue distribution of partiti-like viruses in *S*. *exempta*

We generated standard curves using an absolute quantification method of qPCR for quantification of SEIV1 and SEIV2 (S2 Table; S3 Fig; S1 Data). The concentration of partiti-like viruses used for detection of transmission and host range were 3.8 × 10^8^ copy numbers/μl and 2.7 × 10^7^ copy numbers/μl for SEIV1 and SEIV2, respectively. We then created strains of *S*. *exempta* using single pairs of moths that were not infected by known viruses or *Wolbachia* (tested with transcriptome and specific primers (S1B Fig; S1 Table; S2 Table). Using newly-hatched larvae, we showed that the partiti-like viruses could not be horizontally-transmitted by oral infection but we could infect hosts successfully by microinjection with high efficiency (70%) (Table 1). Vertically, the viruses could be maternally-transmitted to offspring with high efficiency (100%) (Table 1).

**Table 1:** Transmission mode of SEIV1 and SEIV2. Infected individuals = “+”, uninfected individuals = “-”.

Transmission mode	Species	Individuals	Number testing +ve (SEIV1)	Number testing -ve (SEIV1)	Transmission efficiency (%)	Number testing +ve (SEIV2)	Number testing -ve(SEIV2)	Transmission efficiency (%)
Horizontal	*Spodoptera exempta*	Peroral infection	0	30	0	0	30	0
	*S*. *exempta*	Microinjection	21	9	70	21	9	70
	*S*. *frugiperda*	Microinjection	15	5	75	15	5	75
	*S*. *littoralis*	Microinjection	18	10	64.3	18	10	64.3
	*Helicoverpa armigera*	Microinjection	8	8	50	8	8	50
Vertical	*S*. *exempta*	Female+/Male+	24	0	100	24	0	100
	*S*. *exempta*	Female-/Male+	0	24	0	0	24	0
	*S*. *exempta*	Female+/Male-	24	0	100	24	0	100
	*S*. *exempta*	Female-/Male-	0	24	0	0	24	0
	*S*. *frugiperda*	Female+/Male+	24	0	100	24	0	100
	*S*. *frugiperda*	Female-/Male+	0	24	0	0	24	0
	*S*. *frugiperda*	Female+/Male-	24	0	100	24	0	100
	*S*. *frugiperda*	Female-/Male-	0	24	0	0	24	0
	*S*. *littoralis*	Female+/Male+	24	0	100	24	0	100
	*S*. *littoralis*	Female-/Male+	0	24	0	0	24	0
	*S*. *littoralis*	Female+/Male-	24	0	100	24	0	100
	*S*. *littoralis*	Female-/Male-	0	24	0	0	24	0
	*H*. *armigera*	Female+/Male+	2	10	16.7	2	10	16.7

**Fig 2:**
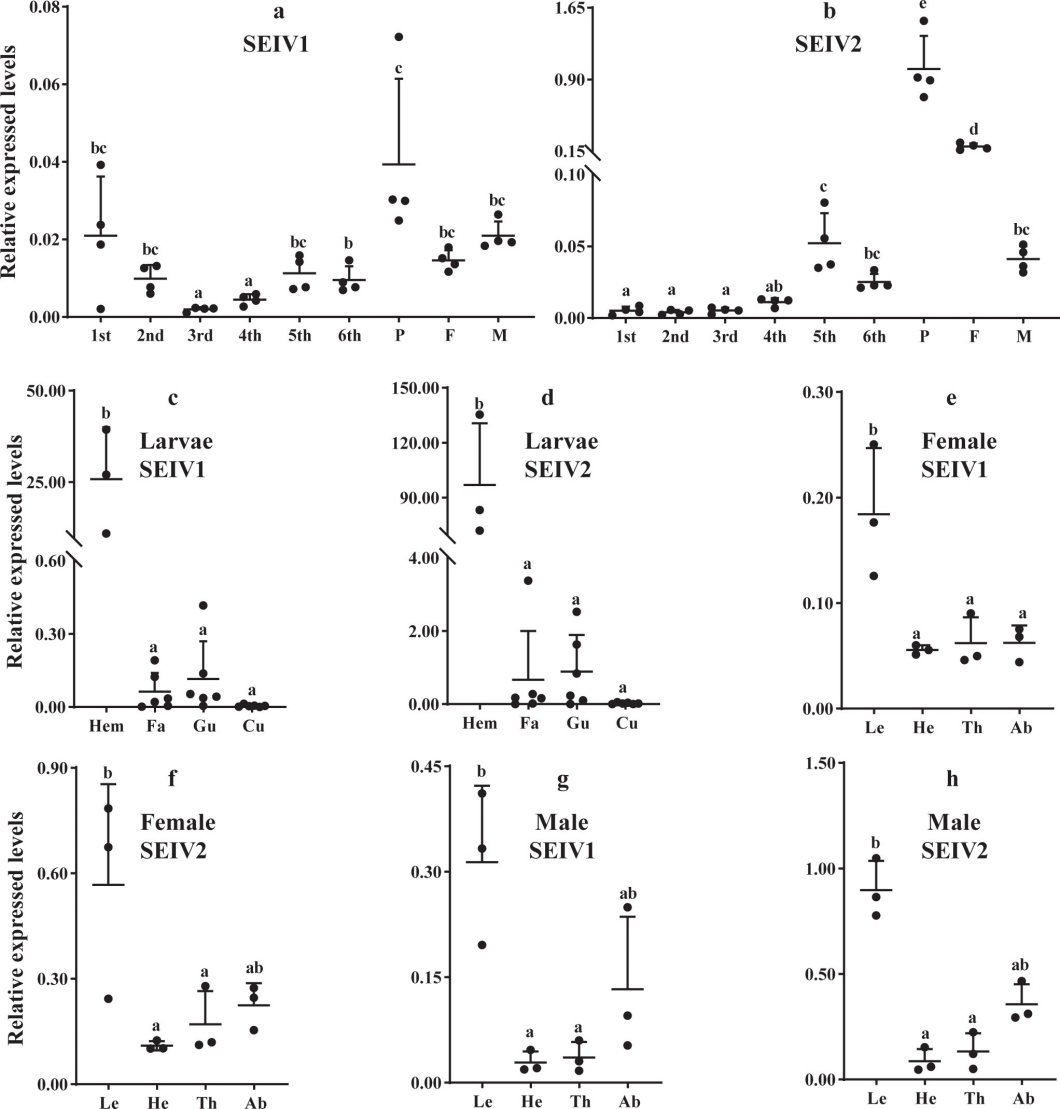
Distribution of the partiti-like viruses in *S*. *exempta*. (a,b) Relative quantification of SEIV1 (a) and SEIV2 (b) using β-actin and GAPDH as reference genes at different stages (n = 4) (ANOVA: SEIV1: F = 10.111, d.f. = 8,27, P < 0.0001; SEIV2: F = 100.73, d.f. = 8,27, P < 0.0001). (c,d) Relative quantification of SEIV1 (c) and SEIV2 (d) in different tissues of larvae (for hemolymph, n = 3; others, n = 6) (SEIV1: F = 26.827, d.f. = 3,17, P < 0.0001; SEIV2: F = 13.819, d.f. = 3,17, P < 0.0001). (e,f) Relative quantification of SEIV1 (e) and SEIV2 (f) in different tissues of female (n = 3) (SEIV1: F = 10.875, d.f. = 3,8, P = 0.0034; SEIV2: F = 6.681, d.f. = 3,8, P = 0.0143). (g,h) Relative quantification of SEIV1 (g) and SEIV2 (h) in different tissues of male (n = 3) (SEIV1: F = 11.114, d.f. = 3,8, P = 0.0032; SEIV2: F = 14.539, d.f. = 3,8, P = 0.0013). 1^st^ = first instar-stage larvae, 2^nd^ = second instar-stage larvae, 3^rd^ = third instar-stage larvae, 4^th^ = fourth instar-stage larvae, 5^th^ = fifth instar-stage larvae, 6^th^ = sixth instar-stage larvae, p = pupae, F = Female, M = Male, Hem = Hemolymph, Fa = Fat body, Gu = Gut, Cu = Cuticle, Le = leg, He = Head, Th = Thorax, Ab = Abdomen. Means ± SD. Different letters indicate statistically significance differences.

We determined the tissue and stage distribution of SEIV1 and SEIV2 in *S*. *exempta* using β-actin and GAPDH as references genes. The patterns for the two viruses were broadly similar, with virus titers (as measured by log-transformed relative gene expression levels) varying significantly across the life-stages, being highest in the pupal stage and lowest in the larval stages (Tukey’s LSD; Fig 2A and 2B; S2 Data). For larvae, there was highly significant variation between body parts in their virus titers, with virus levels being highest in the hemolymph and lowest in the other body parts (Tukey’s LSD; Fig 2C and 2D; S2 Data). Adult females (Tukey’s LSD, Fig 2E and 2F; S2 Data) and males (Tukey’s LSD, Fig 2G and 2H; S2 Data) also exhibited significant variation in virus levels among body parts, generally being highest in the legs and abdomen and lowest in the head and thorax.

### Effect of partiti-like viruses on the development, fecundity and adult longevity of *S*. *exempta*

**Fig 3:**
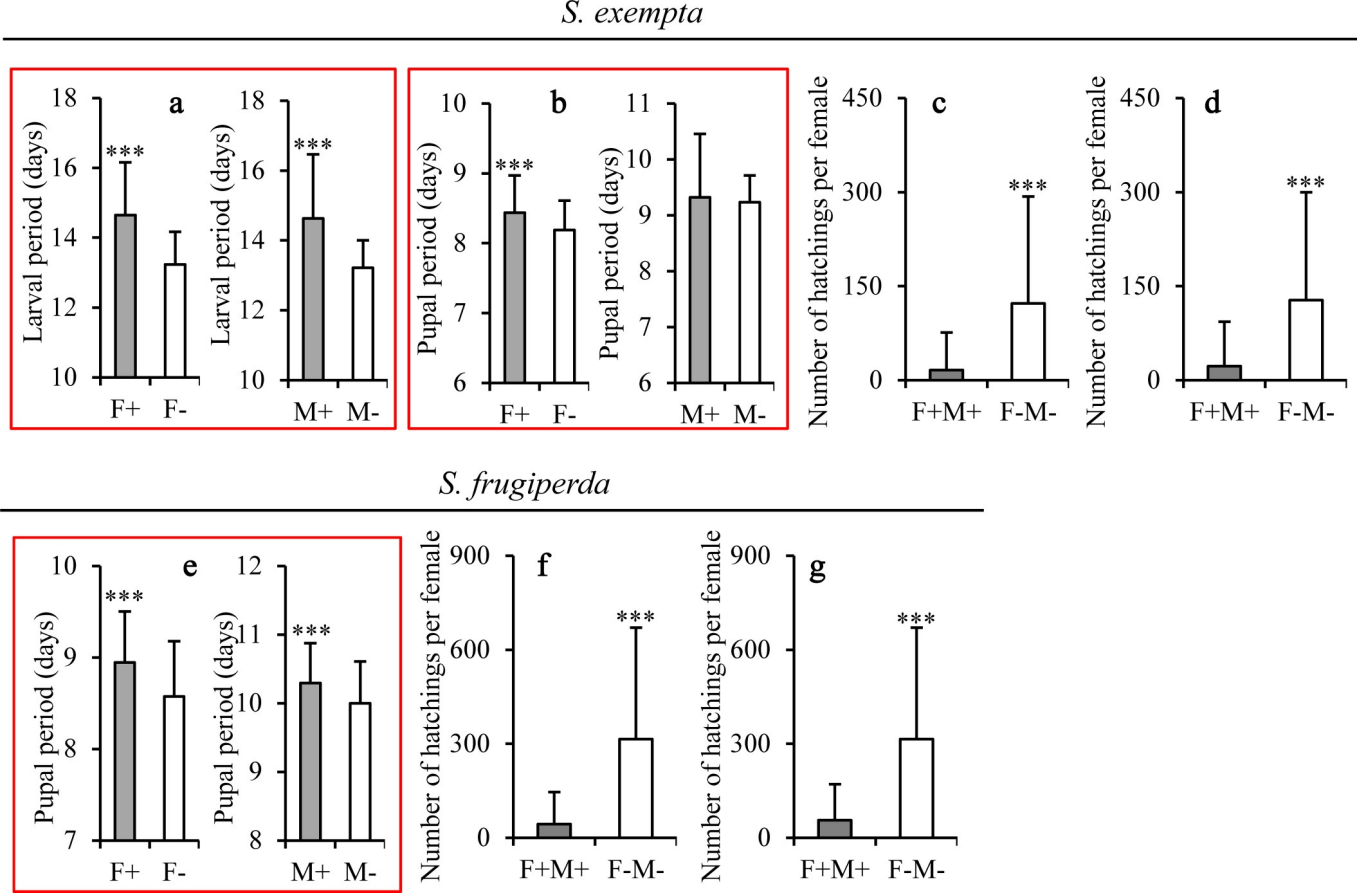
Life-history parameters of the partiti-like viruses-positive and -negative individuals of *S*. *exempta* and *S*. *frugiperda*. (a,b) *S*. *exempta* larval period (a) (females: t-test: t = 8.192, d.f. = 212, P < 0.0001; males: t = 6.550, d.f. = 176, P < 0.0001) and pupal period (b) (females: t = 3.729, d.f. = 212, P = 0.0002; males: t = -0.079, d.f. = 176, P = 0.94 (n: F+ = 114, F- = 100, M+ = 100, M- = 78). (c,d) Fertility (number of neonate larvae produced) of F+M+ and F-M- *S*. *exempta* pairs in all samples (c) (t = -5.031, d.f. = 74, P < 0.0001;n: F+M+ = 61, F-M- = 25) and after excluding pairs that produced no eggs (d) (t = -4.107, d.f. = 58, P = 0.00013; n: F+M+ = 43, F-M- = 24). (e) *S*. *frugiperda* pupal period (females: t = 4.82, d.f. = 225, P < 0.0001; males: t = 3.46, d.f. = 203, P <0.0001; n: females: F+ = 110, F- = 117; males: M+ = 123, M- = 82). (f,g) Fertility (number of neonate larvae produced) of F+M+ and F-M- *S*. *frugiperda* individuals in all samples (f) (t = -4.087, d.f. = 57, P = 0.00014; n: F+M+ = 30, F-M- = 29) or after excluding pairs that produced no eggs (g) (t = -0.978, d.f. = 50, P = 0.0031; n: F+M+ = 23, F-M- = 29). Means ± SD. *** = P<0.001, based on t-tests at each point.

In both female and male *S*. *exempta*, larval development was extended in insects infected with the three partiti-like viruses (Fig 3A; S3 Data). In females, but not males, pupal development was also extended in virus-infected insects (Fig 3B; S3 Data). Pupal weight was not affected by virus infection in either sex (S4A Fig, S3 Data) and neither was adult longevity (S4B Fig; S3 Data).

Infected *S*. *exempta* pairs were significantly more likely to produce no eggs than non-infected pairs (29% vs 4%; logistic regression: χ^2^ = 8.039, d.f. = 1, P = 0.0046), and this translated into significantly lower offspring production for infected than non-infected pairs (Fig 3C; S3 Data). When pairs that produced no eggs were excluded from the analysis, the partiti-like viruses-infected pairs again produced fewer eggs than non-infected pairs (Fig 3D; S3 Data), indicating that the partiti-like viruses infection not only affected fertility but also reduced either the number of eggs laid by egg-laying females or their egg hatch rate.

There was no difference in larval mortality rates between the partiti-like viruses-infected and non-infected larvae, however although marginally fewer infected insects were deformed as pupae, a higher proportion of the partiti-like viruses-infected insects died as pupae (S3 Table; S3 Data). Taken together, the mean estimated *R*_0_ for the partiti-like viruses-infected insects was lower than that for non-infected insects (mean ± SE = 1.209 ± 0.116 *versus* 9.873 ± 0.808).

### Host range of the partiti-like viruses

By single-pair matings of *S*. *frugiperda*, *S*. *littoralis* and *Helicoverpa armigera*, we constructed strains that were free of partiti-like viruses, SEPV, NPV and *Wolbachia* (i.e. below the level of PCR detection using specific primers) (S1B Fig; S2 Table). Horizontally, the partiti-like viruses could infect *S*. *frugiperda*, *S*. *littoralis* and *H*. *armigera* by microinjection but with different infection efficiencies, ranging from 50–75% (Table 1). As with *S*. *exempta*, the efficiency of vertical transmission of SEIV1 and SEIV2 was 100% in *S*. *frugiperda* and *S*. *littoralis*, however the transmission efficiency in the more distantly-related *H*. *armigera* was very low (17%) (Table 1). Absolute quantitative PCR revealed that virus titers did not differ significantly between sodium hypochlorite-treated and non-treated eggs, suggesting that in all three *Spodoptera* species, vertical transmission of these viruses was *transovarial* (within the egg itself) rather than *transovum* (due to virus contamination of the eggshell) (S5 Fig;S1 Data)

### Effect of partiti-like viruses on the development, fecundity and adult longevity of *S*. *frugiperda*

To determine the effect of *S*. *exempta*-derived partiti-like viruses on its congeneric *S*. *frugiperda*, first we used a transcriptome and PCR analysis to establish that there were no viruses or *Wolbachia* present in the *S*. *frugiperda* laboratory population used in the bioassays (S1 Table; S2 Table). As with *S*. *exempta*, in both females and males, *S*. *frugiperda* pupal development was extended in the partiti-like viruses-infected insects (Fig 3E, S4 Data). However, infection with the partiti-like viruses had no effect on larval development, pupal weight or adult longevity of either sex (S4C–S4E Fig; S4 Data).

As with *S*. *exempta*, when *S*. *frugiperda* moth pairs were infected with the partiti-like viruses, they were significantly more likely to produce no eggs than non-infected pairs (23% vs 0%; logistic regression: χ^2^ = 10.381, d.f. = 1, P = 0.0013). This translated into significantly lower offspring production for infected pairs (Fig 3F, S4 Data), even when those that produced no eggs were excluded from the analysis (Fig 3G; S4 Data), indicating again that the partiti-like viruses infection not only affected fertility but also either the number or hatch-rate of eggs laid by egg-laying females.

There was no significant difference in the larval mortality rates of the partiti-like viruses-infected and non-infected larvae, however although marginally more infected insects were deformed as pupae, a smaller proportion of the partiti-like viruses-infected insects died as pupae (S4 Table; S4 Data). Taken together, the mean estimated *R*_0_ for the partiti-like viruses-infected insects was significantly lower than that for non-infected insects (mean ± SE = 0.570 ± 0.056 *versus* 9.718 ± 0.659).

### Interaction between the partiti-like viruses and NPV in two *Spodoptera* species

**Fig 4:**
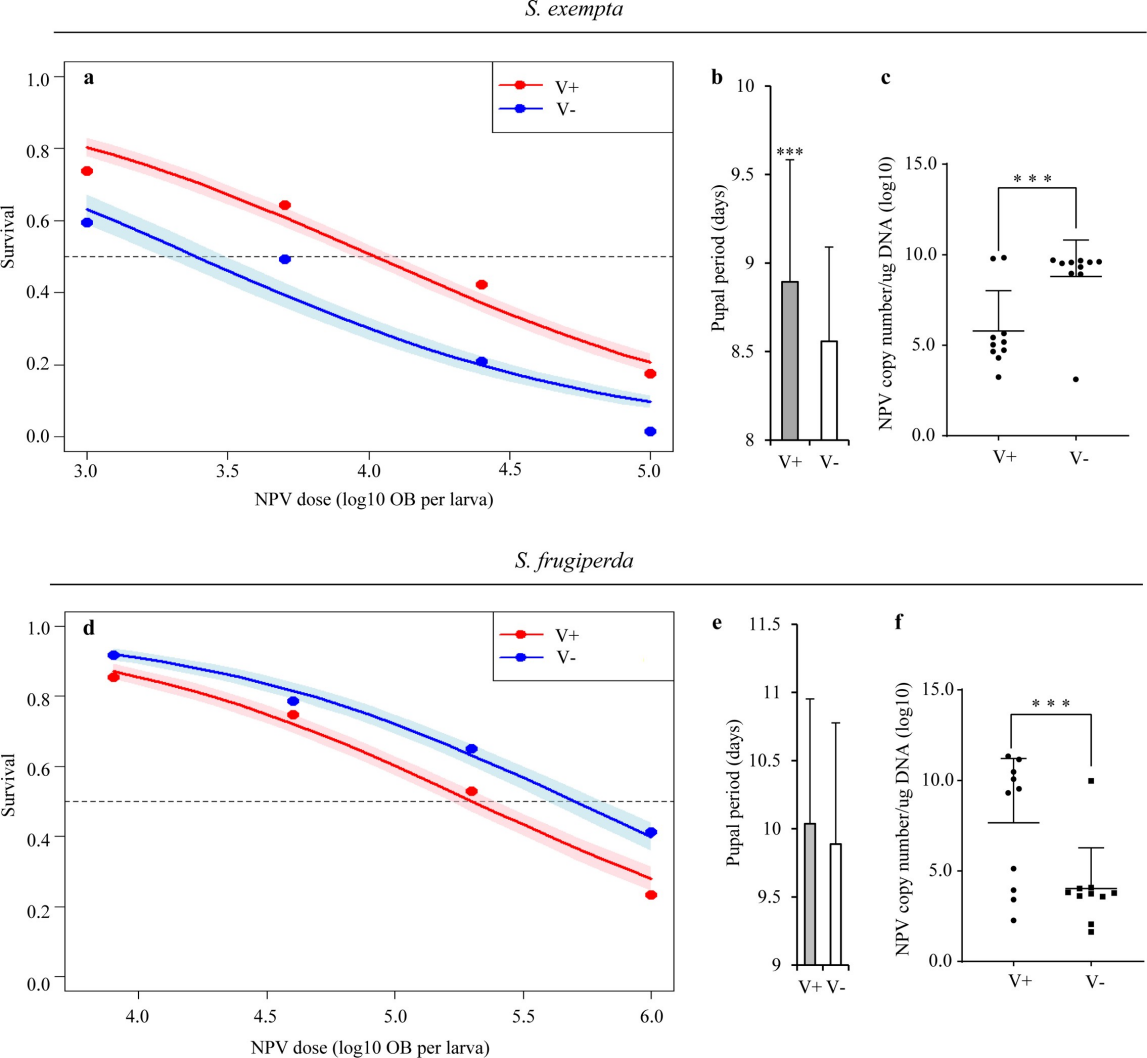
Relationship between the baculoviruses (SpexNPV and SfMNPV) and the partiti-like viruses in larvae of *S*. *exempta* (a-c) and *S*. *frugiperda* (d-f). (a,d) Effect of NPV dose (log_10_-transformed number of occlusion bodies per larva) on larval survival to pupation in *S*. *exempta* (a) (logistic regression: χ^2^ = 25.798, d.f. = 1, P < 0.0001) and *S*. *frugiperda* (d) (logistic regression: χ^2^ = 9.447, d.f. = 1, P = 0.0022). The points are the raw means for each dose. The thick lines are the fitted values and the shaded zones are the standard errors around these fitted values; blue points, lines and shading are the three partiti-like viruses-negative larvae (V-); red points, lines and shading are the three partiti-like viruses-positive larvae (V+). The numbers of larvae at different concentrations for SpexNPV (0 (control), 1×10^3^, 5×10^3^, 2.5×10^4^, and 1×10^5^ OBs/larva) were 63, 64, 67, 82, 79 for virus-negative individuals and 96, 109, 134, 133, 127 for virus-negative individuals; for SfMNPV (0 (control), 8×10^3^, 4×10^4^, 2×10^5^, and 1×10^6^ OBs/larva) they were 92, 85, 104, 149, 87 for virus-negative individuals and 100, 95, 111, 106, 90 for virus-positive individuals. (b,e) The pupal period of survivors of NPV-challenge in *S*. *exempta* (b) (linear model: F = 12.263, d.f. = 1,265, P = 0.00054) and *S*. *frugiperda* (e) (linear model: F = 2.30, d.f. = 1,413, P = 0.0940). (c,f) NPV copy numbers (log_10_-transformed) at 72 h post-challenge with SpexNPV (c) (F = 10.095, d.f. = 18, P = 0.0052) and SfMNPV (f) (F = 7.504, d.f. = 18, P = 0.0134). The concentrations of NPV were 2.5×10^4^ OBs/larva and 2×10^5^ OBs/larva for SpexNPV and SfMNPV respectively. V- = the three partiti-like viruses-negative larvae, V+ = the three partiti-like viruses-positive larvae. Means ± SD. *** = P<0.001, based on t-tests at each time-point.

In *S*. *exempta*, larvae that hosted the partiti-like viruses were significantly more resistant to SpexNPV challenge than those that lacked the partiti-like viruses (logistic regression: χ^2^ = 25.798, d.f. = 1, P < 0.0001; Fig 4A; S5 Data). In addition, and as expected, NPV-induced mortality increased with virus concentration and there was no interaction between NPV concentration and viral status (log10 NPV dose: χ^2^ = 155.539, d.f. = 1, P < 0.0001; interaction term: χ^2^ = 1.349, d.f. = 1, P = 0.247). Survivors of NPV challenge had significantly longer pupal development periods if they carried the partiti-like viruses than if they did not (linear model: F = 12.263, d.f. = 1,265, P = 0.00054; Fig 4B; S5 Data); the effects of NPV dose and the interaction between NPV and partiti-like virus were both non-significant (log_10_ NPV concentration: F = 1.437, d.f. = 1,264, P = 0.0683; interaction term: F = 0.240, d.f. = 1,263, P = 0.455).

In *S*. *frugiperda*, larvae that carried the partiti-like viruses were significantly more susceptible to SfMNPV challenge than those that lacked the partiti-like viruses (logistic regression: χ^2^ = 9.447, d.f. = 1, P = 0.0022; Fig 4D; S6 Data); in addition, NPV-induced mortality increased with viral dose, as expected, and there was no interaction between NPV dose and viral status (log10 NPV dose: χ^2^ = 134.407, d.f. = 1, P < 0.0001; interaction term: χ^2^ = 0.492, d.f. = 1, P = 0.483). The pupal development period of survivors of NPV-challenge was not affected by whether or not they harbored the partiti-like viruses (linear model: F = 2.30, d.f. = 1,413, P = 0.0940; Fig 4E; S6 Data); NPV concentration and the interaction between NPV and partiti-like viruses were also both non-significant (log_10_ NPV conc: F = 1.04, d.f. = 1,412, P = 0.2593; interaction term: F = 0.000, d.f. = 1,411, P = 0.975).

We tested the differences in the NPV replication rate at 72 h post-challenge between the partiti-like viruses-positive and -negative individuals by repeating the bioassay with 2.5 × 10^4^ OBs/larva and 2 × 10^5^ OBs/larva for SpexNPV and SfMNPV, respectively. The titer of NPV (as measured by absolute expression levels; S3 Fig) differed across the four species-virus combinations (ANOVA: F = 6.632, d.f. = 3,36, P = 0.0011). This was due to a significant interaction between host species and partiti-like viruses status (two-way ANOVA: host species: F = 3.137, d.f. = 1,36, P = 0.0850; virus-status: F = 0.1453, d.f. = 1,36, P = 0.705; interaction term: F = 16.614, d.f. = 1,36, P = 0.00024). Specifically, when infected with partiti-like viruses, the NPV load decreased for *S*. *exempta* (Fig 4C; S7 Data), but increased for *S*. *frugiperda* (Fig 4F; S7 Data).

### Transcriptome analysis in *S*. *exempta* and *S*. *frugiperda*

**Fig 5:**
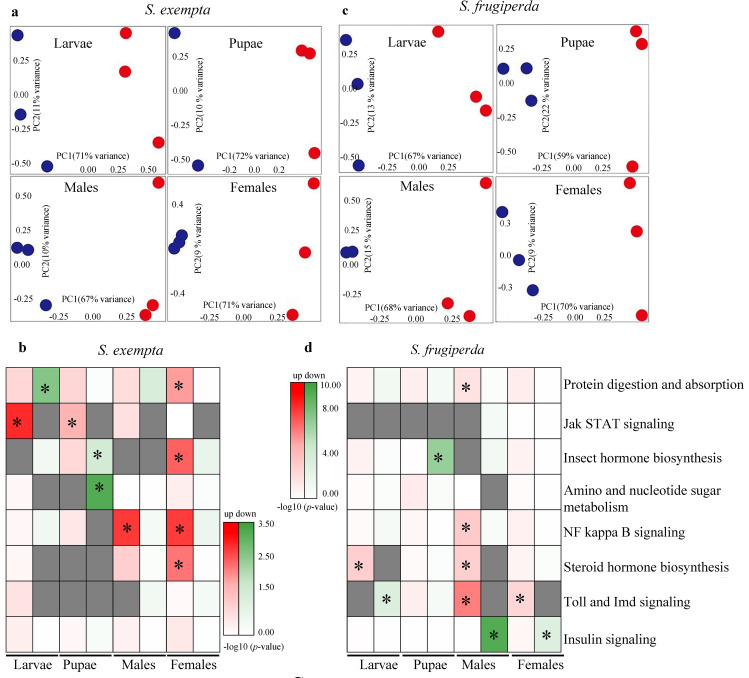
Transcriptome analysis using the partiti-like virus-positive individuals compared to related virus-negative individuals in *S*. *exempta* and *S*. *frugiperda*. (a,c) PCA analysis of global gene expression of DEGs in the comparison of partiti-like virus-positive groups and related virus-negative groups in *S*. *exempta* (a) and *S*. *frugiperda* (c). Blue stands for the partiti-like viruses-positive samples and red stands for the partiti-like viruses-negative samples. (b,d) Heatmaps of–log10 *p*-values of KEGG pathway representing the up- and down- regulated DEGs in *S*. *exempta* (b) and *S*. *frugiperda* (d). “*” indicate the significantly enriched pathways (p < 0.05). Red color shows up-regulation pathways, green color show down-regulation pathways, gray color shows no value, the redder/greener the color, the lower *P*-values.

To understand the dynamic interaction between the partiti-like viruses and their hosts, we determined the differentially-expressed genes (DEGs) between the transcriptomes of partiti-like viruses-positive and -negative individuals at different life stages of *S*. *exempta* and *S*. *frugiperda*, including larvae, pupae, and adult males and females (S1 Table; S5 Table). We chose several unigenes to validate their expression levels by qPCR (S6 Fig; S7 Fig; S2 Table; S1 Data). Interestingly, in both *S*. *exempta* and *S*. *frugiperda*, the number of DEGs was higher in adult females than that in other life-stages, suggesting that these viruses play a more important role in adult females than in other life-stages (S8 Fig; S8 Data). The principal component analysis (PCA) with DEG data clearly distinguished partiti-like viruses-positive from -negative individuals at different stages in the two species (Fig 5A and 5C; S9 Data). Taken together with the hierarchical clustering of these DEGs (S9 Fig; S10 Fig; S1 Data), these results suggest that the partiti-like viruses have a major effect on the gene expression profiles of their hosts.

**Fig 6:**
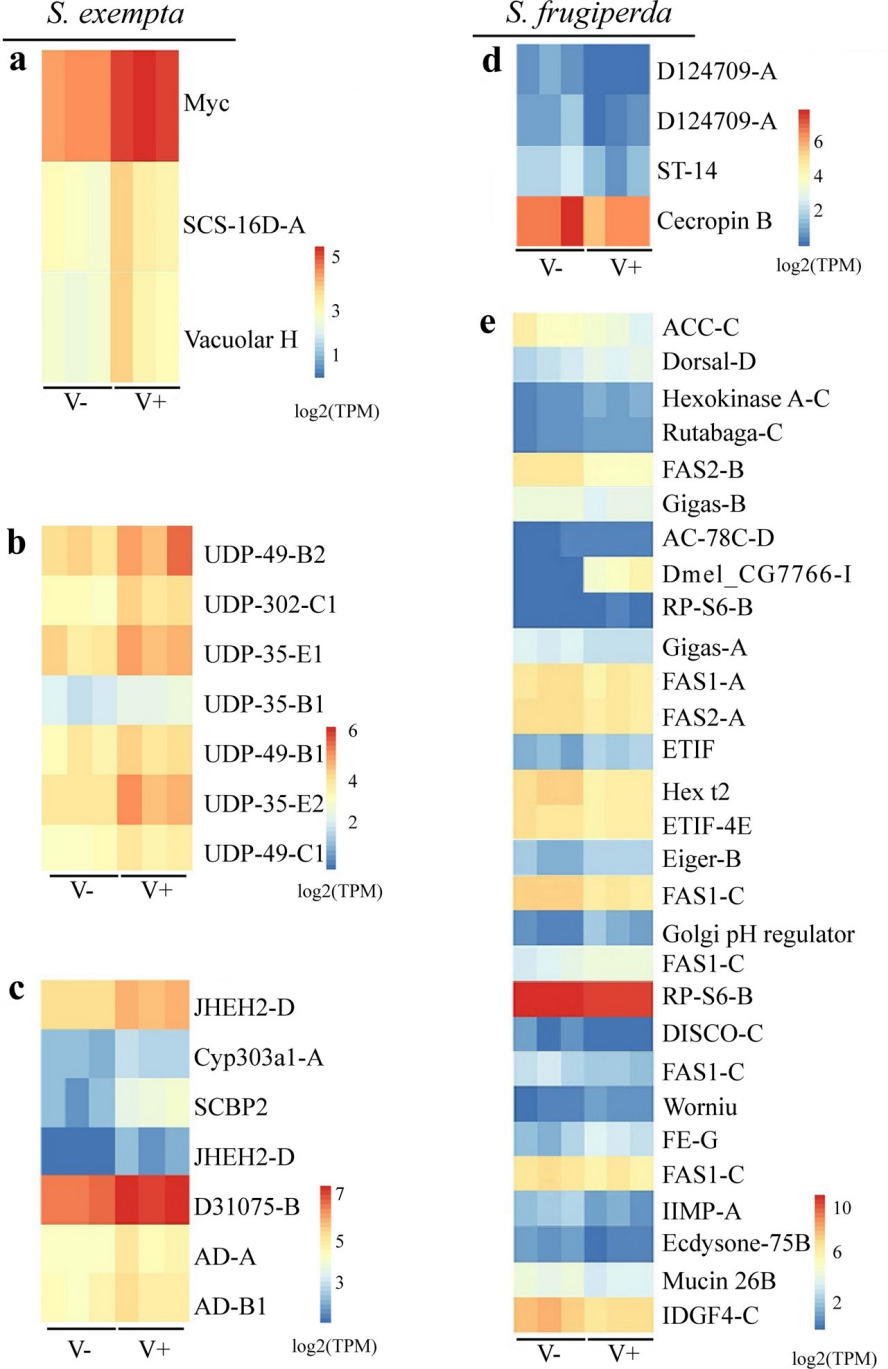
The quantity of DEGs with log2(TPM) related to the expression of different pathways. (a) the Jak-STAT signaling pathway of *S*. *exempta* larvae; (b) the Insect hormone biosynthesis pathway of *S*. *exempta* adult females; (c) the Steroid hormone biosynthesis pathway of *S*. *exempta* adult female; (d) the Toll and Imd signaling pathway of *S*. *frugiperda* larvae; and (e) the insulin signaling pathway expression of *S*. *frugiperda* adult females. Colors in log2(TPM) indicate the gene expression levels, the hotter (redder) the color, the higher the gene expression level. The original data for TPM are shown in S9 Data.

We performed pathway enrichment analysis on the DEGs, focusing particular attention to pathways related to the development, immune and reproduction systems (Fig 5B and 5D; S9 Data; S10 Data). Interestingly, genes in Jak-STAT immune signaling pathway, which are related to some antiviral mechanisms, were significantly enriched and up-regulated only in the partiti-like viruses-infected larvae and pupae of *S*. *exempta* (Fig 5B, Fig 6A; S9 Data), but not in any life stages of *S*. *frugiperda* (Fig 5D; S9 Data). Genes in the insect hormone biosynthesis pathway, which are related to the degradation of Juvenile Hormone (JH) (Fig 5B, Fig 6B; S9 Data), and genes in the steroid hormone biosynthesis pathway, which prevent ecdysone functioning and are related to the reproduction of adult females, were significantly enriched and up-regulated in the partiti-like viruses-infected adult female *S*. *exempta* (Fig 5B, Fig 6C; S9 Data), suggesting that the partiti-like viruses may impact *S*. *exempta* reproduction via these two pathways. Genes in the Toll and Imd signaling pathway, which are related to immune response of hosts mainly against bacteria, were significantly down-regulated at the larval stage in *S*. *frugiperda* infected with the partiti-like viruses (Fig 5D, Fig 6D; S9 Data), with no comparable differential gene expression in *S*. *exempta*. The insulin signaling pathway, the genes of which are related to female reproduction, were significantly down-regulated in adult female *S*. *frugiperda* infected by the partiti-like viruses (Fig 5D, Fig 6E; S9 Data), suggesting that female reproduction may be regulated by different pathways in these two species.

## Discussion

The development of next-generation sequencing technologies has facilitated the recent discovery of novel viruses that do not present obvious symptoms of infection in their hosts [1,2,3,4,5]. In the present study, we used RNA-seq, to search for new viruses in *S*. *exempta*, a major crop pest in sub-Saharan Africa. Usually, the RNA-seq method can only be used to find viruses with a poly(A) tail or poly(A) structure in their genome (RNA viruses) or transcripts (DNA viruses), but we cannot exclude the possibility that non-poly(A) viruses may also be present. We discovered three new partiti-like viruses, and quantified the impact of these viruses on both *S*. *exempta* and a novel host, *S*. *frugiperda*, which has recently been introduced to the same geographical range as *S*. *exempta* [27]. We show that in both species the partiti-like viruses are vertically-transmitted with high efficiency but could be horizontally-transmitted only via microinjection. The partiti-like viruses reduced the growth rate and fecundity of both species, but their impact on susceptibility to nucleopolyhedrovirus challenge differed across the two species, reducing the susceptibility of its native host (*S*. *exempta*) but increasing the susceptibility of its new host (*S*. *frugiperda*).

To determine the presence of possibly harmful viruses in *S*. *exempta*, we chose newly-collected insects from the field, but also tested samples from a long-term lab colony. Indeed, we found new viruses only in field-collected individuals, most likely because their negative impacts on host fitness saw them purged from our lab colony [48]. We also sequenced a population of *S*. *frugiperda* that had been reared in the lab for more than 20 generations, but failed to detect any new viruses. Phylogenetic analysis of the metagenome [49] revealed that the novel viruses clustered with partiti-like viruses (*Partitiviridae*). Members of *Partitiviridae* are known viruses of plants, fungi and protists and the first record from an insect (*Drosophila*) was only in 2015 [5], with subsequent reports in other invertebrates also using NGS approaches [1,5,37]. The genomes of partiti-like viruses typically contain two double-stranded RNAs: RNA1 encodes polypeptides for virus-replication containing RdRp domain and RNA2 encodes a capsid protein [37,50]. Here, we only report the whole genome of RNA1, as we failed to annotate RNA2 due to a lack of referable conserved domains [1].

Endosymbionts of insects are usually transmitted via maternal inheritance although they can be horizontally transmitted [11,51]. However, many viruses can be efficiently transmitted both vertically and horizontally by oral infection [12,52,53,54,55]. Interestingly, we found that in *S*. *exempta*, the partiti-like viruses were efficiently transmitted both vertically (via transovarial maternal inheritance) and horizontally, but horizontal transmission was possible only by microinjection. This suggests that in the field, the partiti-like viruses are likely to be almost exclusively transmitted vertically from parent to offspring, in much the same way as the *S*. *exempta* endosymbiotic bacterium, *Wolbachia* [9,56]. By microinjection, we showed that the partiti-like viruses could horizontally-infect *S*. *frugiperda*, *S*. *littoralis* and *H*. *armigera* with similar efficiencies. Vertical transmission was also high in *S*. *exempta*, *S*. *frugiperda* and *S*. *littoralis*, but not in the more distantly-related *H*. *armigera*. Parasitoids and ectoparasites can act as vectors to transmit some viruses horizontally between insect hosts, e.g. deformed wing virus (DWV) is transmitted by *Varroa destructor* in honeybees, and polydnavirus (PDV) is transmitted by wasps in lepidopteran species [57,58]. Therefore, we cannot exclude the possibility that partiti-like viruses could be horizontally-transmitted within or among species naturally, though this remains to be tested. If natural horizontal transmission can occur via this route, then it is possible that these partiti-like viruses might naturally jump from *S*. *exempta* to *S*. *frugiperda* now that their ranges overlap across much of sub-Saharan Africa, following the recent introduction of *S*. *frugiperda* to the region [23,27].

Viruses form diverse evolutionary relationships with their hosts, from parasitic, e.g. by killing them directly (e.g. baculoviruses) [6,10], to mutualistic, by increasing their host’s fitness though a variety of means [12,59], or they may be conditionally mutualistic, by being either beneficial or harmful to their host depending on context, including which other symbionts are present in the holobiome [60,61]. In our system, the partiti-like viruses decreased the growth rate of larvae and the reproduction of adults in their original host, *S*. *exempta*. Since many novel infectious diseases emerge via host-jump events [62,63,64], we transinfected the partiti-like viruses into *S*. *frugiperda*, to establish whether they would have similar effects on a novel host. In *S*. *frugiperda*, the partiti-like viruses decreased the growth rate of pupae and reproduction of adults significantly, suggesting a potential capacity for controlling *S*. *frugiperda* via maternal inheritance of the virus. Managed host-shifts of microbes have been reported in insects previously, e.g. the endosymbiotic bacterium *Wolbachia* has been used to block mosquito-borne disease transmission, and insect-pathogenic viruses have been used for pest management [8,65,66,67]. But as far as we are aware, this is the first time this approach has been considered using novel viruses detected by next-generation sequencing approaches.

The baculoviruses are considered an important group of pathogens for developing commercial biopesticides against lepidopteran crop pests, contributing to a 5% share of the global pesticide market [6,7]. Studies exploring the interaction between baculoviruses and other microbes, such as *Wolbachia* and HaDV2, have offered interesting insights into how baculovirus effectiveness might be enhanced or restricted in the field, which is necessary for improving their use and exploiting novel viruses as viable biopesticides [9,12]. Heritable symbionts are considered to be maintained in populations primarily through bringing benefits or manipulating reproduction [68,69]. In *S*. *exempta*, the partiti-like viruses were maternally-transmitted but decreased host reproduction, suggesting a negative selection pressure of these viruses in populations of *S*. *exempta*. However, the partiti-like viruses also increased the resistance of *S*. *exempta* to SpexNPV, a baculovirus that is extremely prevalent in natural populations of *S*. *exempta* [9,70]. This defines a possible role for the partiti-like viruses in their host’s life-history, and ultimately may promote their prevalence in field populations. Interestingly, when we initiated a host-shift event to *S*. *frugiperda*, the partiti-like viruses infection had the effect of *increasing* their host’s susceptibility SfMNPV, a virus that is currently widely used as a commercial biopesticide to combat fall armyworm.

Transcriptome analysis is a useful tool for exploring the interaction between such microbes and their hosts [71,72]. To collect more evidence supporting the interaction between the partiti-like viruses and the two *Spodoptera* species, we performed RNA-seq with larvae, pupae and adults. Due to the effects of the partiti-like viruses on their hosts, we focused on pathways related to immunity, development and reproduction [73,74,75,76,77,78]. In *S*. *exempta* larvae, only the Jak-STAT pathway was significantly enriched by up-regulation in the partiti-like viruses-positive 5^th^-instar larvae. In fruit flies and mosquitoes, this pathway controls the expression of antiviral genes in response to infection with a range of viruses including *Drosophila* C virus, dengue virus and West Nile virus [79]; its role in lepidopteran antiviral immunity is not yet so well established, though it has been implicated in immune responses against NPV in the silkworm moth, *Bombyx mori* [80,81]. This is consistent with our NPV bioassay results, which showed that partiti-like viruses significantly increased the resistance of *S*. *exempta* to SpexNPV [75,77]. No antiviral pathways were enriched in virus-positive or -negative 5^th^-instar *S*. *frugiperda*, however, the Toll and Imd immune signaling pathway, which is also involved in insect immune responses [71,77], was significantly down-regulated in *S*. *frugiperda* larvae. These results are consistent with those of the NPV bioassays, which showed that larvae infected with partiti-like viruses had significantly greater susceptibility to SfMNPV.

We also observed that fertility was markedly reduced in adult *S*. *exempta* and *S*. *frugiperda* when infected with the partiti-like viruses. Hormones, including JH and ecdysone, are essential for reproduction in insects [78]. Interestingly, the transcriptome data suggest that the partiti-like viruses could decrease these hormone levels in female *S*. *exempta* by up-regulation of the expression levels of genes related to the degradation of JH and the inactivation of ecdysteroid hormone. Insulin pathways have vital roles in insect reproduction as nutritional sensors, regulating reproductive tissues and the biosynthesis of JH and ecdysteroids [78]. Moreover, the insulin signaling pathway in female *S*. *frugiperda* were significantly down-regulated, suggesting that the partiti-like viruses may reduce the fertility of *S*. *exempta* and *S*. *frugiperda* via different pathways. Certainly, these initial expression study results open up some interesting avenues for more research into the role of partiti-like viruses on host immune and reproduction pathways.

## Conclusion

In summary, our studies highlight the potentially important virus interactions that can occur in different hosts. In the original host, *S*. *exempta*, the partiti-like viruses reduce its host’s growth rate and reproduction, but also enhance its resistance to the host-specific baculovirus, SpexNPV, suggesting a conditional mutualistic relationship and possible virus-host co-evolution in field populations. Interestingly, in *S*. *frugiperda*, these partiti-like viruses reduced host reproduction and decreased larval resistance to SfMNPV, illustrating a potential prospective use for managing *S*. *frugiperda*. New technologies now make the detection of novel virus species easier and allow new insights into the co-evolution between viruses and their original host, as well as the interaction between viruses and their novel hosts during host-shifts. Illuminating the function of such viruses may offer novel insights for future pest management strategies.

## Supporting information

S1 Fig PCR detection of the viruses in *Spodoptera exempta*. a) PCR detection of the viruses the partiti-like viruses (SEIV1, SEIV2, SEIV3) and the plant-fungal virus-like virus (SEPV) in *S*. *exempta* larvae. The numbers stand for individual *S*. *exempta* larvae. The SEPV was negative in all detected samples. (b) Construction of the partiti-like viruses negative strains by single pair matings. 2 μg total RNA were used to synthesize cDNA template of 25 μl per sample and 2 μl cDNA was used per PCR reaction. 1 and 2 = female and male of *S*. *exempta*, 3 and 4 = female and male of *S*. *frugiperda*, 5 and 6 = female and male of *S*. *littoralis*, 7 and 8 = female and male of *Helicoverpa armigera*. + = positive control.
(TIF)

S2 Fig Genome sequence of the three partiti-like viruses found in *Spodoptera exempta*. (a-c) The genome sequence by 3’ RACE (a) and 5’ RACE (b) and structure (c) of SEIV1. (d-f) The genome sequence by 3’ RACE (d) and 5’ RACE (e) and structure (f) of SEIV2. (g-i) The genome sequence by 3’ RACE (g) and 5’ RACE (h) and structure (i) of SEIV3. Bold blue line with arrow stands for Open reading frame (ORF). RdRp = RNA dependent RNA polymerase.
(TIF)

S3 Fig The standard curves for absolute quantification of SEIV1 (a), SEIV2 (b), SpexNPV (c) and SfMNPV (d).
(TIF)

S4 Fig Life-history parameters of the partiti-like viruses positive and negative individuals of *S*. *exempta* and *S*. *frugiperda*. (a,b) Parameters of pupal weight (a) (females: t = 1.879, d.f. = 212, P = 0.0616, males: t = 1.473, d.f. = 176, P = 0.143) (females: F+ = 114, F- = 100; males: M+ = 100, M- = 78) and adult longevity (b) (females: t = -0.054, d.f. = 92, P = 0.96, males: t = -0.22, d.f. = 91, P = 0.83) (females: F+ = 61, F- = 33; males: M+ = 60, M- = 33) in *S*. *exempta*. (c-e) Parameters of larval period (c) (t-test: females: t = 0.123, d.f. = 225, P = 0.902; males: t = -0.766, d.f. = 203, P = 0.445), pupal weight (d) (females: t = 0.312, d.f. = 225, P = 0.756, males: t = 0.153, d.f. = 203, P = 0.879) (females: F+ = 110, F- = 117; males: M+ = 123, M- = 82) and adult longevity (e) (females: t = 1.212, d.f. = 57, P = 0.23, males: t = 1.777, d.f. = 57, P = 0.081) (females: F+ = 30, F- = 29; males: M+ = 30, M- = 29) in *S*. *frugiperda*. Means ± SD. *** = P<0.001, based on t-tests at each point.
(TIF)

S5 Fig Absolute quantification of SEIV1 and SERIV2 in eggs. In *S*. *exempta*, SEIV1: t = 1.094, d.f. = 6, P = 0.3160, SEIV2: t = 0.5417, d.f. = 6, P = 0.6075). In *S*. *frugiperda*, SEIV1: t = 1.110, d.f. = 6, P = 0.3096, SEIV2: t = 0.7930, d.f. = 6, P = 0.4580. W = eggs watched with 5% sodium hypochlorite, C = control. Statistics was done with unpaired t-test. Means ± SD.
(TIF)

S6 Fig Detection of different expressed genes (DEGs) generated by RNA-seq with sybgreen qPCR in *S*. *exempta* using β-actin and GAPDH as reference genes. (a-c) DEGs from larvae (for a: t = 4.697, d.f. = 4, P = 0.0093; for b: t = 10.513, d.f. = 4, P = 0.0005; for c: t = 4.655, d.f. = 4, P = 0.0096). (d-f) DEGs from pupae (for d: t = 62.440, d.f. = 4, P < 0.0001; for e: t = 38.447, d.f. = 4, P < 0.0001; for f: t = 7.162, d.f. = 4, P = 0.0020). (g-i) DEGs from males (for g: t = 9.052, d.f. = 4, P = 0.0008; for h: t = 6.359, d.f. = 4, P = 0.0031; for i: t = 4.049, d.f. = 4, P = 0.0155). (j-o) DEGs from females (for j: t = 5.729, d.f. = 4, P = 0.0046; for k: t = 2.959, d.f. = 4, P = 0.0416; for l: t = 3.716, d.f. = 4, P = 0.0205; for m: t = 8.283, d.f. = 4, P = 0.0012; for n: t = 2.997, d.f. = 4, P = 0.0400; for o: t = 2.094, d.f. = 4, P = 0.0364). The contig numbers were shown. Statistics was done with unpaired t-test. Means ± SD. “*” stand for P < 0.05, “**” stand for P < 0.001, “***” stand for P < 0.001.
(TIF)

S7 Fig Detection of different expressed genes (DEGs) generated by RNA-seq with sybgreen qPCR in *S*. *frugiperda* using β-actin and GAPDH as reference genes. (a-c) DEGs from larvae (for a: t = 4.348, d.f. = 4, P = 0.0122; for b: t = 5.649, d.f. = 4, P = 0.0048; for c: t = 3.465, d.f. = 4, P = 0.0257). (d-f) DEGs from pupae (for d: t = 5.267, d.f. = 4, P = 0.0062; for e: t = 3.358, d.f. = 4, P = 0.0277; for f: t = 6.314, d.f. = 4, P = 0.0032). (g,h) DEGs from males (for g: t = 3.902, d.f. = 4, P = 0.0175; for h: t = 2.816, d.f. = 4, P = 0.0480). (i-l) DEGs from females (for i: t = 5.773, d.f. = 4, P = 0.0045; for j: t = 3.565, d.f. = 4, P = 0.0235; for k: t = 7.728, d.f. = 4, P = 0.0015; for l: t = 27.477, d.f. = 4, P < 0.0001). The contig numbers were shown. Statistics was done with unpaired t-test. Means ± SD. “*” stand for P < 0.05, “**” stand for P < 0.001, “***” stand for P < 0.001.
(TIF)

S8 Fig DEGs numbers using the partiti-like viruses-positive individuals compared to related -negative individuals (V+ vs V-) in *S. exempta* (a) and *S. frugiperda* (b).Numbers above zero in Y axis stand for DEGs that are up-regulated and those below zero stand for the ones are down-regulated.
(TIF)

S9 Fig The heatmap shows the dynamic change of DEGs expression in response to partiti-like viruse infection at different stages in *S. exempta*, including larvae (a), pupae (b), male (c) and female (d). The color key representing the log10 TPM values for each unigene. Red indicates a higher gene expression, blue indicates lower expression. “V+” stand for samples infected by the partiti-like viruses, “V-” stand for negative samples.
(TIF)

S10 Fig The heatmap shows the dynamic change of DEGs expression in response to partiti-like viruse infection at different stages in *S. frugiperda*, including larvae (a), pupae (b), male (c) and female (d). The color key representing the log10 TPM values for each unigene. Red indicates a higher gene expression, blue indicates lower expression. “V+” stand for samples infected by the partiti-like viruses, “V-” stand for negative samples.
(TIF)

S1 Table Host information and data output for each pool of caterpillar samples. The accession numbers of samples generated by RNA-seq.
(DOCX)

S2 Table Primers used in this study. (DOCX)

S3 Table The influence of partiti-like viruses on survival rates of *S*. *exempta*. (DOCX)

S4 Table The influence of partiti-like viruses on survival rates of *S*. *frugiperda*. (DOCX)

S5 Table The description of transcriptome in the two *Spodoptera* species. (DOCX)

S1 Text The genome sequence of three partiti-like viruses and SEPV. (TXT)

S2 Text Sequences used for tree-based analysis. (TXT)

S1 Data The original data for supplementary figures, including S3 Fig, S5 Fig, S6 Fig, S7 Fig, S9 Fig and S10 Fig. (XLSX)

S2 Data The original data for tissue and stage distribution of SEIV1 and SEIV2. The data were generated with qPCR using β-actin and GAPDH as reference genes.
(XLSX)

S3 Data The original data of life parameters of *S*. *exempta*. The data, including development, body weight and fecundity, were used for Fig 3, S4 Fig and S3 Table.
(XLSX)

S4 Data The original data of life parameters of *S*. *frugiperda*. The data, including development, body weight and fecundity, were used for Fig 3, S4 Fig and S4 Table.
(XLSX)

S5 Data The original data of NPV bioassay with *S*. *exempta*. The data were used to generate Fig 4A and Fig 4B.
(XLSX)

S6 Data The original data of NPV bioassay with *S*. *frugiperda*. The data were used to generate Fig 4D and Fig 4E.
(XLSX)

S7 Data The original data of NPV copy numbers in *S*. *exempta* and *S*. *frugiperda*. The data were obtained with qPCR by standard curve method for generating S4C Fig and S4F Fig.
(XLSX)

S8 Data The significantly differentially expressed genes in the two *Spodoptera* species. All significantly expressed genes were shown and used for S8 Fig, including the annotation and TPM values of the genes in each samples.
(XLSX)

S9 Data The original data used in Fig 5 and Fig 6. The data from RNA-seq using samples from the two *Spodoptera* species, include the TPM values of significantly expressed Genes for Fig 5A and 5C, Fig 6A–6E and p values of pathways selected for Fig 5B and 5D.
(XLSX)

S10 Data The significantly enriched pathways in the two *Spodoptera* species. (XLSX)
