# Supplementary figures and images for: Novel partiti-like viruses are conditional mutualistic symbionts in their normal lepidopteran host, African armyworm, but parasitic in a novel host, Fall armyworm
Source: PLoS Pathog. 2020 Jun 22;16(6):e1008467. doi: 10.1371/journal.ppat.1008467 (PMC7332103; doi:10.1371/journal.ppat.1008467)

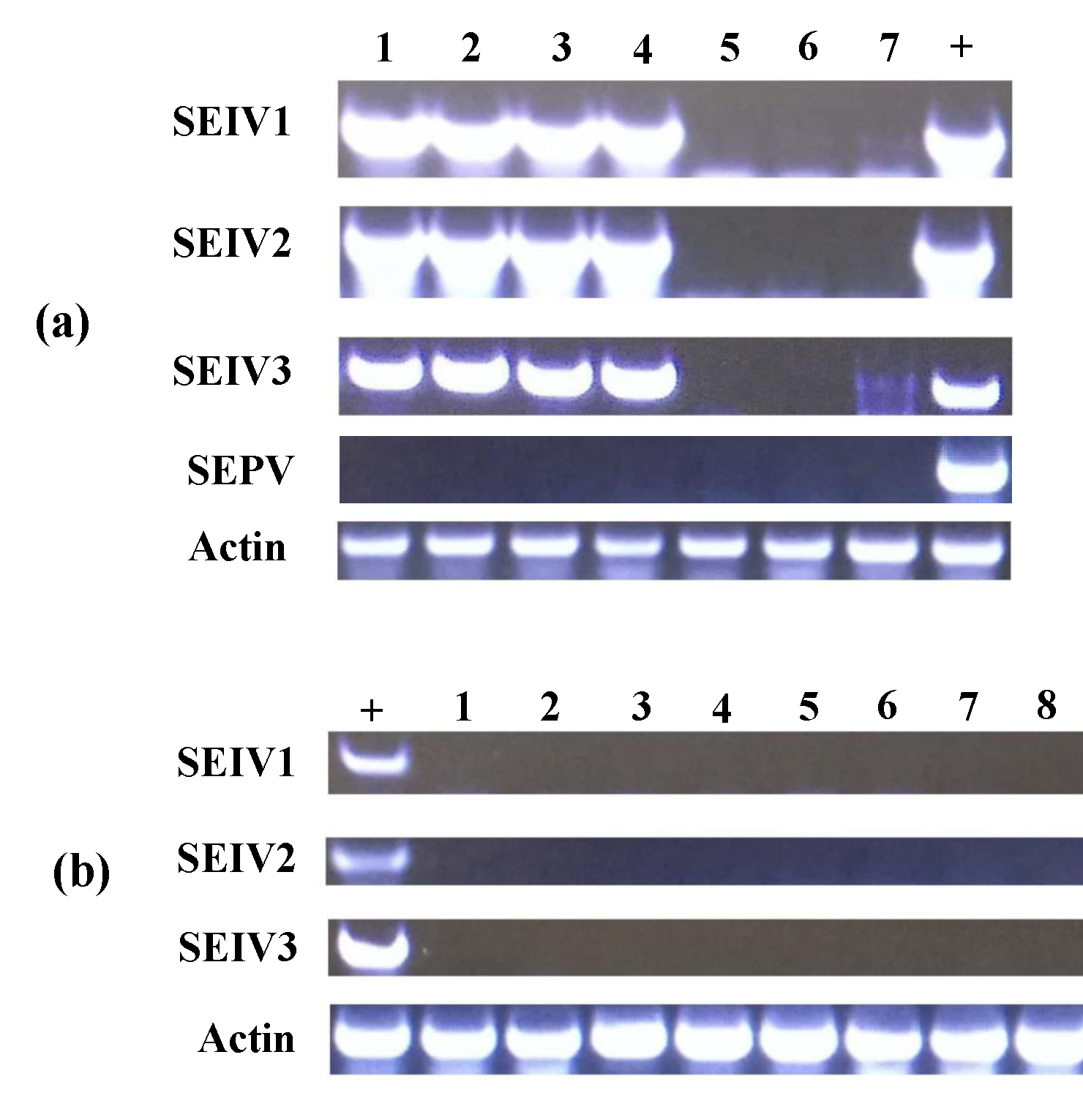

Supplement: S1 Fig — a) PCR detection of the viruses the partiti-like viruses (SEIV1, SEIV2, SEIV3) and the plant-fungal virus-like virus (SEPV) in S. exempta larvae. The numbers stand for individual S. exempta larvae. The SEPV was negative in all detected samples. (b) Construction of the partiti-like viruses negative strains by single pair matings. 2 μg total RNA were used to synthesize cDNA template of 25 μl per sample and 2 μl cDNA was used per PCR reaction. 1 and 2 = female and male of S. exempta, 3 and 4 = female and male of S. frugiperda, 5 and 6 = female and male of S. littoralis, 7 and 8 = female and male of Helicoverpa armigera. + = positive control. (TIF) [file ppat.1008467.s001.tif]

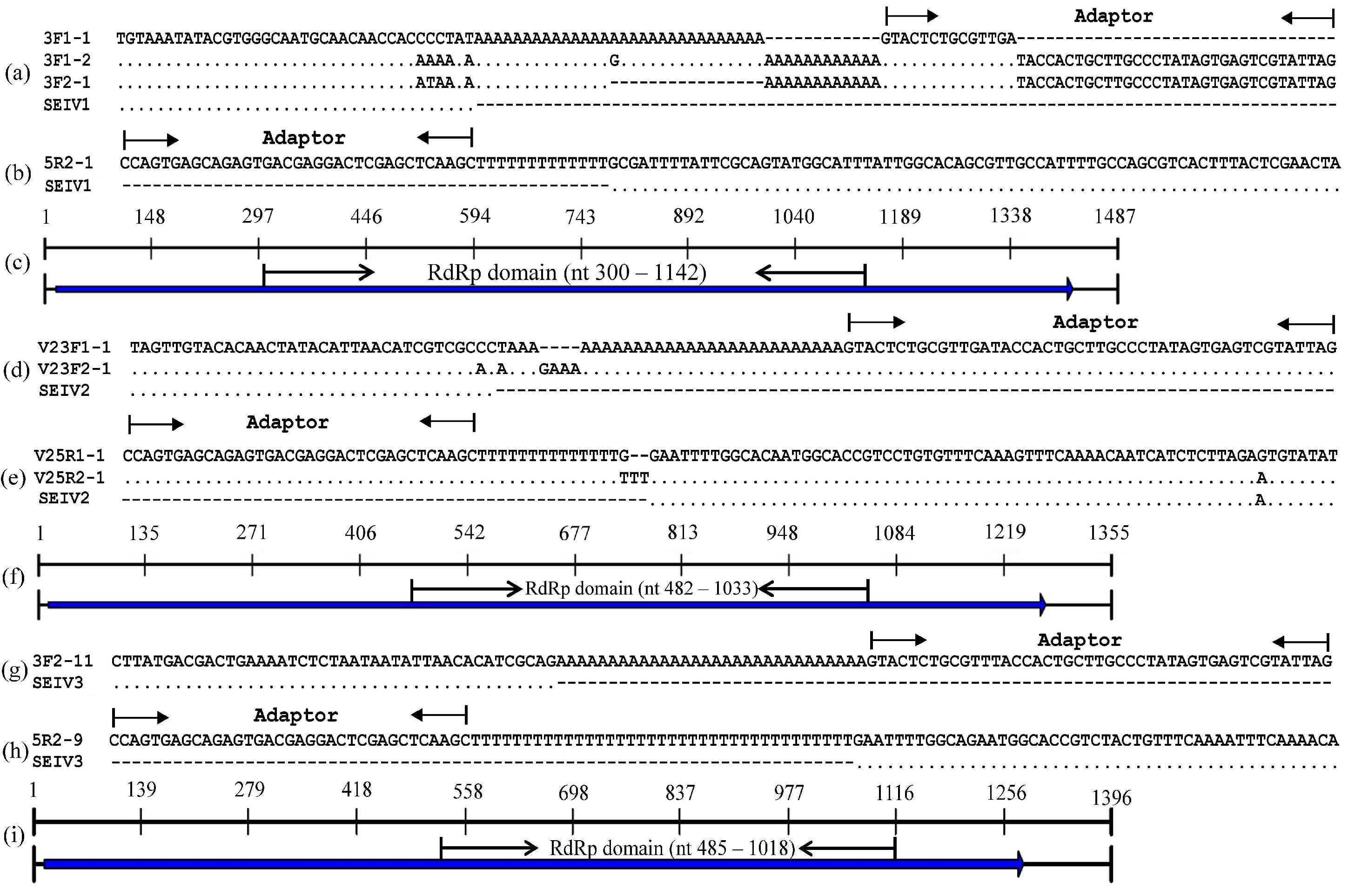

Supplement: S2 Fig — (a-c) The genome sequence by 3’ RACE (a) and 5’ RACE (b) and structure (c) of SEIV1. (d-f) The genome sequence by 3’ RACE (d) and 5’ RACE (e) and structure (f) of SEIV2. (g-i) The genome sequence by 3’ RACE (g) and 5’ RACE (h) and structure (i) of SEIV3. Bold blue line with arrow stands for Open reading frame (ORF). RdRp = RNA dependent RNA polymerase. (TIF) [file ppat.1008467.s002.tif]

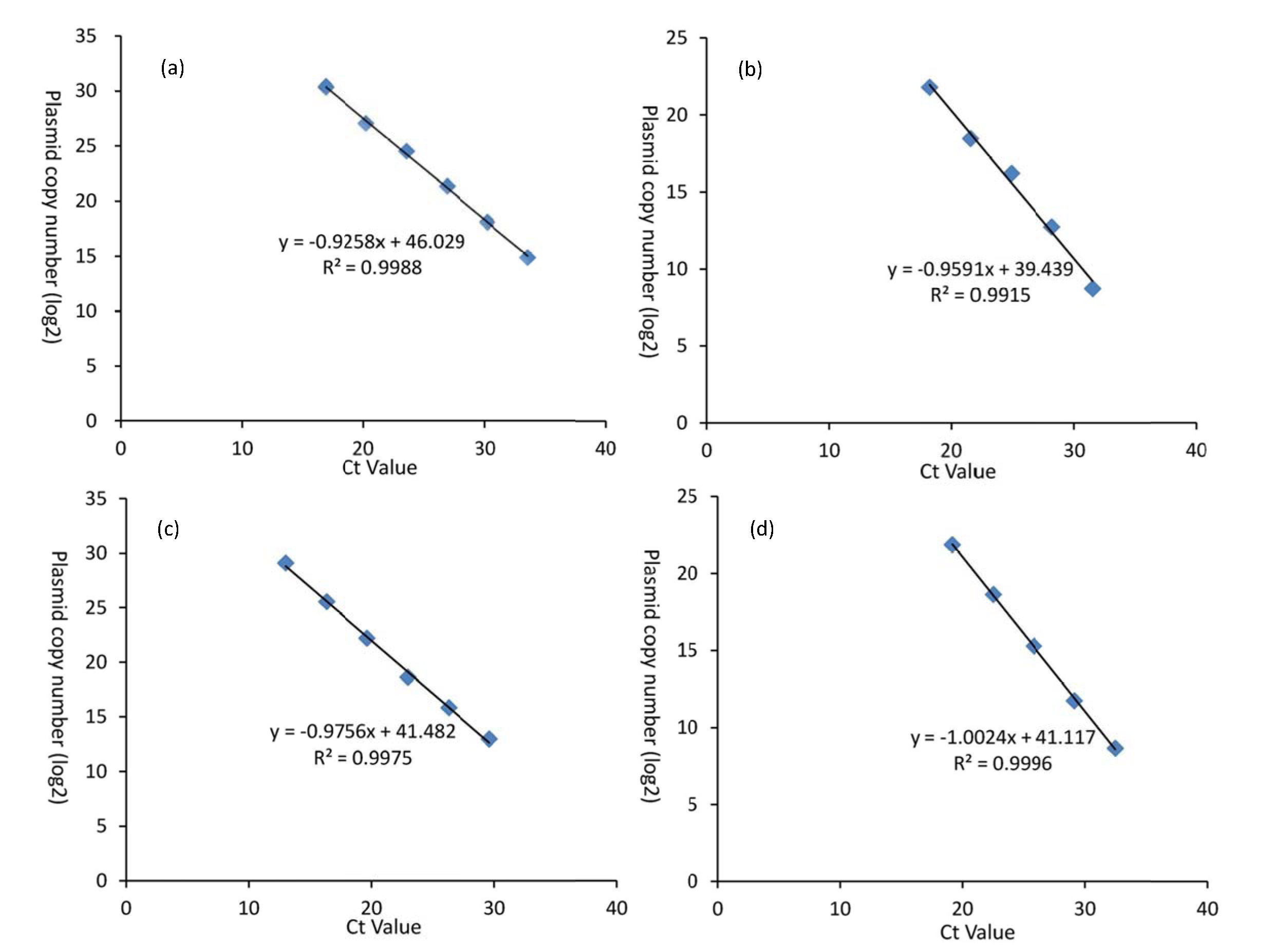

Supplement: S3 Fig — The standard curves for absolute quantification of SEIV1 (a), SEIV2 (b), SpexNPV (c) and SfMNPV (d). (TIF) [file ppat.1008467.s003.tif]

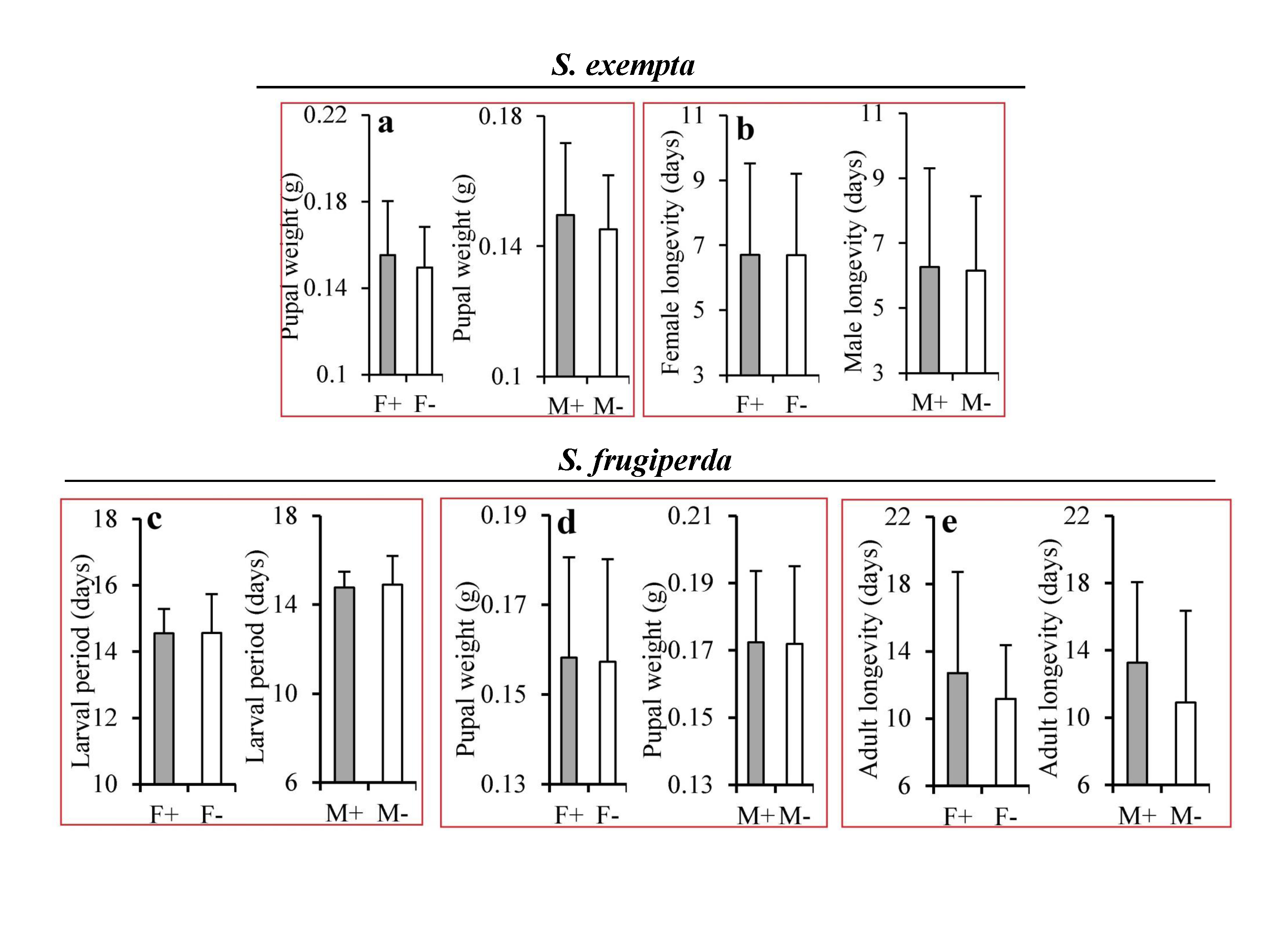

Supplement: S4 Fig — (a,b) Parameters of pupal weight (a) (females: t = 1.879, d.f. = 212, P = 0.0616, males: t = 1.473, d.f. = 176, P = 0.143) (females: F+ = 114, F- = 100; males: M+ = 100, M- = 78) and adult longevity (b) (females: t = -0.054, d.f. = 92, P = 0.96, males: t = -0.22, d.f. = 91, P = 0.83) (females: F+ = 61, F- = 33; males: M+ = 60, M- = 33) in S. exempta. (c-e) Parameters of larval period (c) (t-test: females: t = 0.123, d.f. = 225, P = 0.902; males: t = -0.766, d.f. = 203, P = 0.445), pupal weight (d) (females: t = 0.312, d.f. = 225, P = 0.756, males: t = 0.153, d.f. = 203, P = 0.879) (females: F+ = 110, F- = 117; males: M+ = 123, M- = 82) and adult longevity (e) (females: t = 1.212, d.f. = 57, P = 0.23, males: t = 1.777, d.f. = 57, P = 0.081) (females: F+ = 30, F- = 29; males: M+ = 30, M- = 29) in S. frugiperda. Means ± SD. *** = P<0.001, based on t-tests at each point. (TIF) [file ppat.1008467.s004.tif]

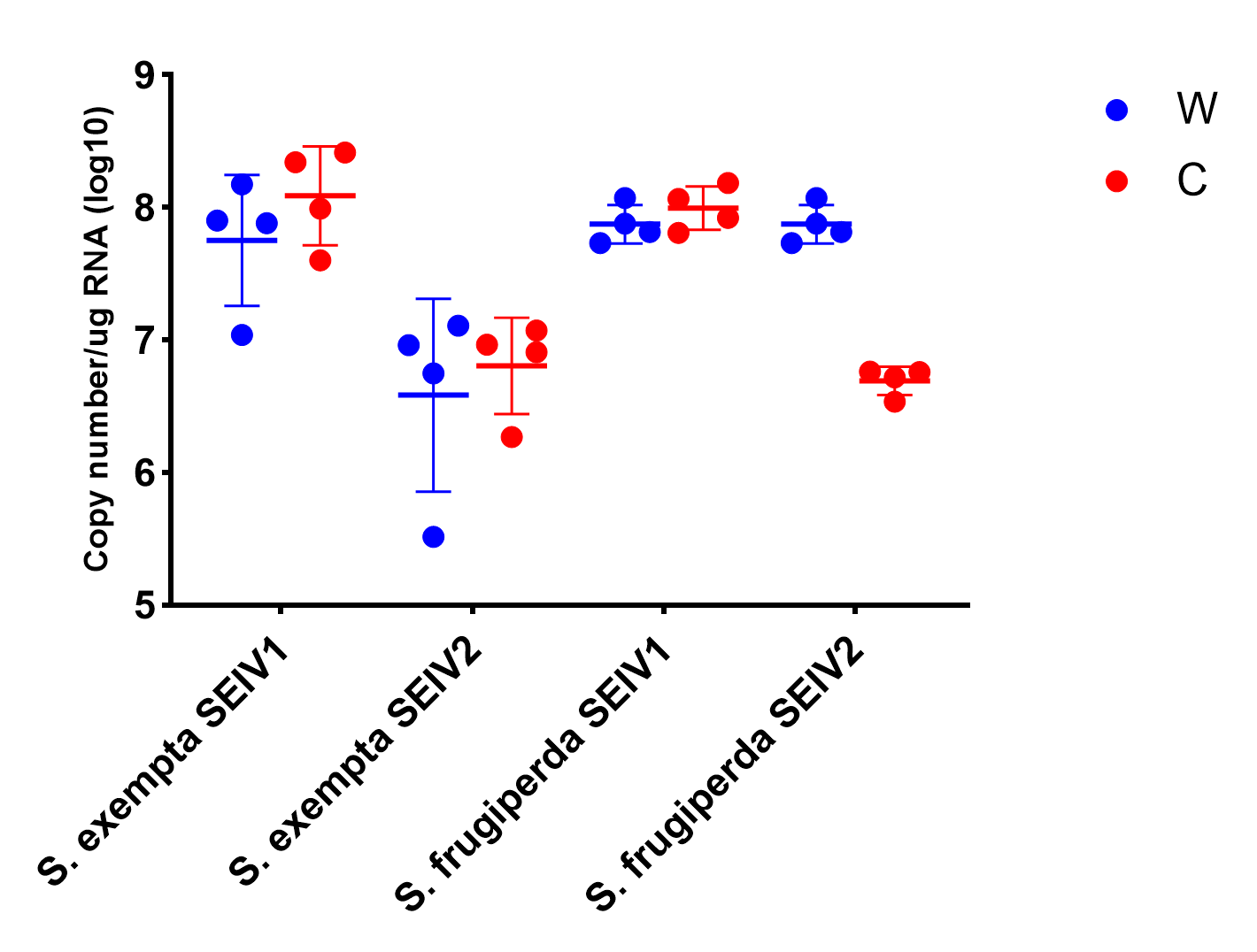

Supplement: S5 Fig — In S. exempta, SEIV1: t = 1.094, d.f. = 6, P = 0.3160, SEIV2: t = 0.5417, d.f. = 6, P = 0.6075). In S. frugiperda, SEIV1: t = 1.110, d.f. = 6, P = 0.3096, SEIV2: t = 0.7930, d.f. = 6, P = 0.4580. W = eggs watched with 5% sodium hypochlorite, C = control. Statistics was done with unpaired t-test. Means ± SD. (TIF) [file ppat.1008467.s005.tif]

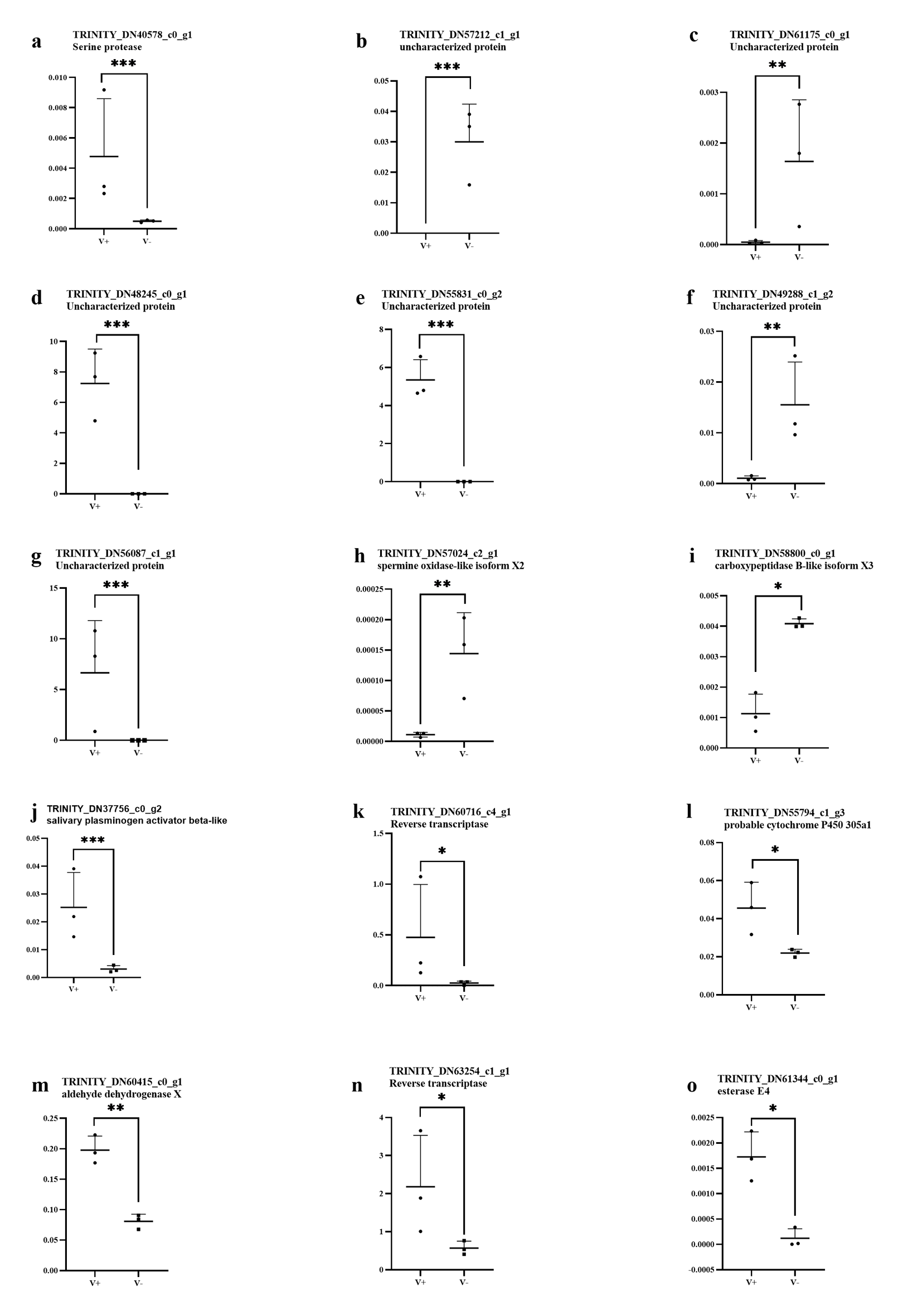

Supplement: S6 Fig — (a-c) DEGs from larvae (for a: t = 4.697, d.f. = 4, P = 0.0093; for b: t = 10.513, d.f. = 4, P = 0.0005; for c: t = 4.655, d.f. = 4, P = 0.0096). (d-f) DEGs from pupae (for d: t = 62.440, d.f. = 4, P < 0.0001; for e: t = 38.447, d.f. = 4, P < 0.0001; for f: t = 7.162, d.f. = 4, P = 0.0020). (g-i) DEGs from males (for g: t = 9.052, d.f. = 4, P = 0.0008; for h: t = 6.359, d.f. = 4, P = 0.0031; for i: t = 4.049, d.f. = 4, P = 0.0155). (j-o) DEGs from females (for j: t = 5.729, d.f. = 4, P = 0.0046; for k: t = 2.959, d.f. = 4, P = 0.0416; for l: t = 3.716, d.f. = 4, P = 0.0205; for m: t = 8.283, d.f. = 4, P = 0.0012; for n: t = 2.997, d.f. = 4, P = 0.0400; for o: t = 2.094, d.f. = 4, P = 0.0364). The contig numbers were shown. Statistics was done with unpaired t-test. Means ± SD. “*” stand for P < 0.05, “**” stand for P < 0.001, “***” stand for P < 0.001. (TIF) [file ppat.1008467.s006.tif]

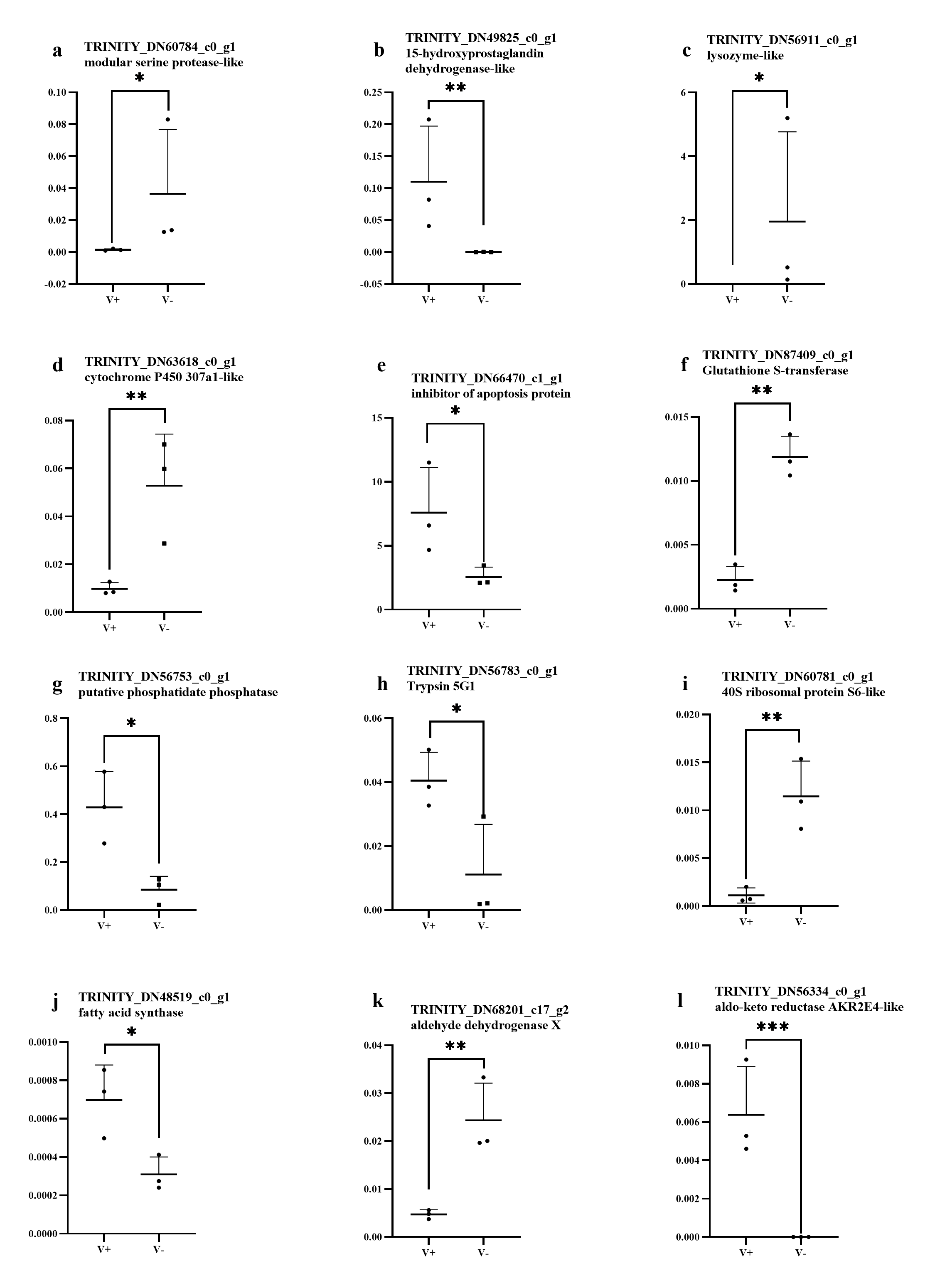

Supplement: S7 Fig — (a-c) DEGs from larvae (for a: t = 4.348, d.f. = 4, P = 0.0122; for b: t = 5.649, d.f. = 4, P = 0.0048; for c: t = 3.465, d.f. = 4, P = 0.0257). (d-f) DEGs from pupae (for d: t = 5.267, d.f. = 4, P = 0.0062; for e: t = 3.358, d.f. = 4, P = 0.0277; for f: t = 6.314, d.f. = 4, P = 0.0032). (g,h) DEGs from males (for g: t = 3.902, d.f. = 4, P = 0.0175; for h: t = 2.816, d.f. = 4, P = 0.0480). (i-l) DEGs from females (for i: t = 5.773, d.f. = 4, P = 0.0045; for j: t = 3.565, d.f. = 4, P = 0.0235; for k: t = 7.728, d.f. = 4, P = 0.0015; for l: t = 27.477, d.f. = 4, P < 0.0001). The contig numbers were shown. Statistics was done with unpaired t-test. Means ± SD. “*” stand for P < 0.05, “**” stand for P < 0.001, “***” stand for P < 0.001. (TIF) [file ppat.1008467.s007.tif]

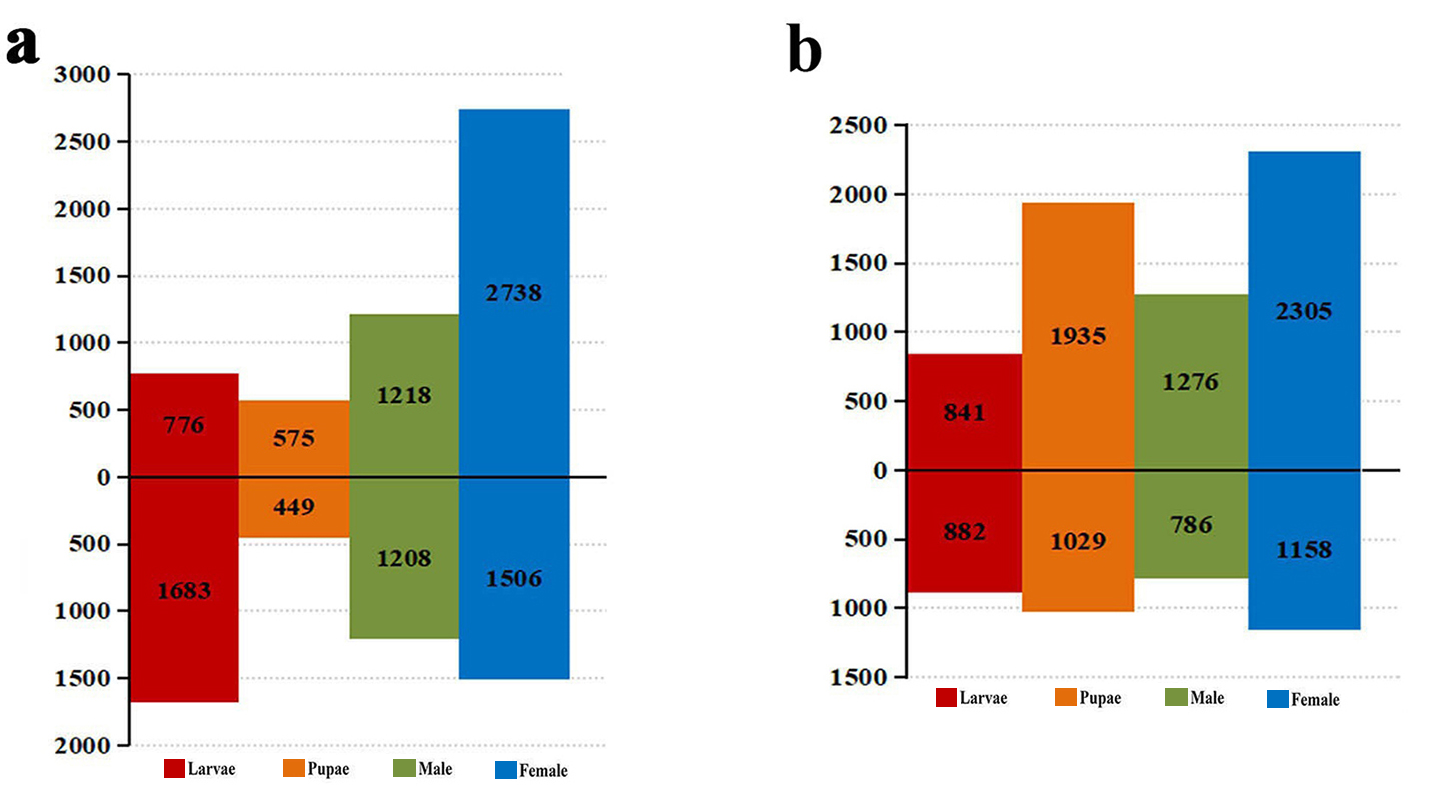

Supplement: S8 Fig — DEGs numbers using the partiti-like viruses-positive individuals compared to related -negative individuals (V+ vs V-) in S. exempta (a) and S. frugiperda (b).Numbers above zero in Y axis stand for DEGs that are up-regulated and those below zero stand for the ones are down-regulated. (TIF) [file ppat.1008467.s008.tif]

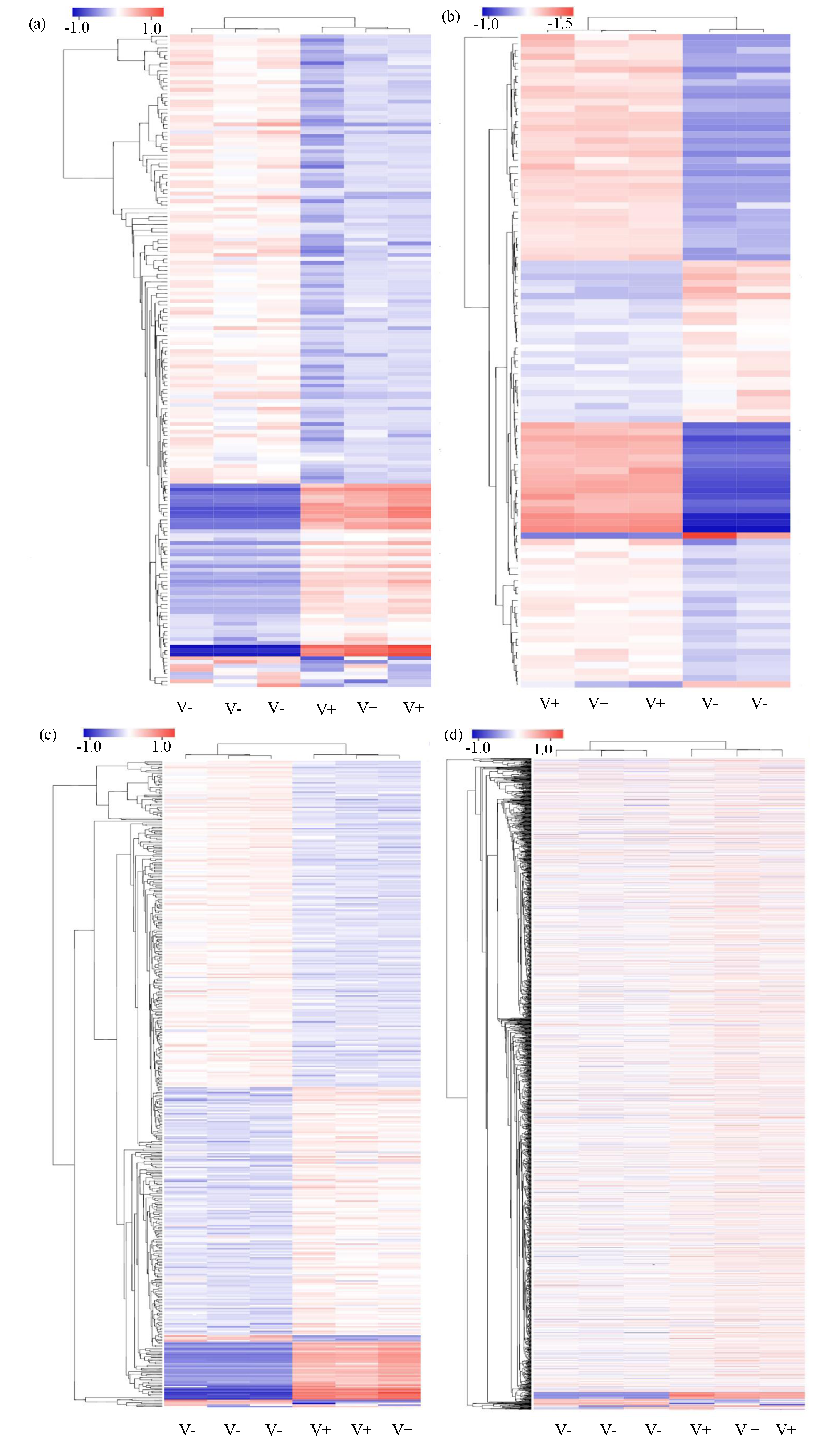

Supplement: S9 Fig — The heatmap shows the dynamic change of DEGs expression in response to partiti-like viruse infection at different stages in S. exempta, including larvae (a), pupae (b), male (c) and female (d). The color key representing the log10 TPM values for each unigene. Red indicates a higher gene expression, blue indicates lower expression. “V+” stand for samples infected by the partiti-like viruses, “V-” stand for negative samples. (TIF) [file ppat.1008467.s009.tif]

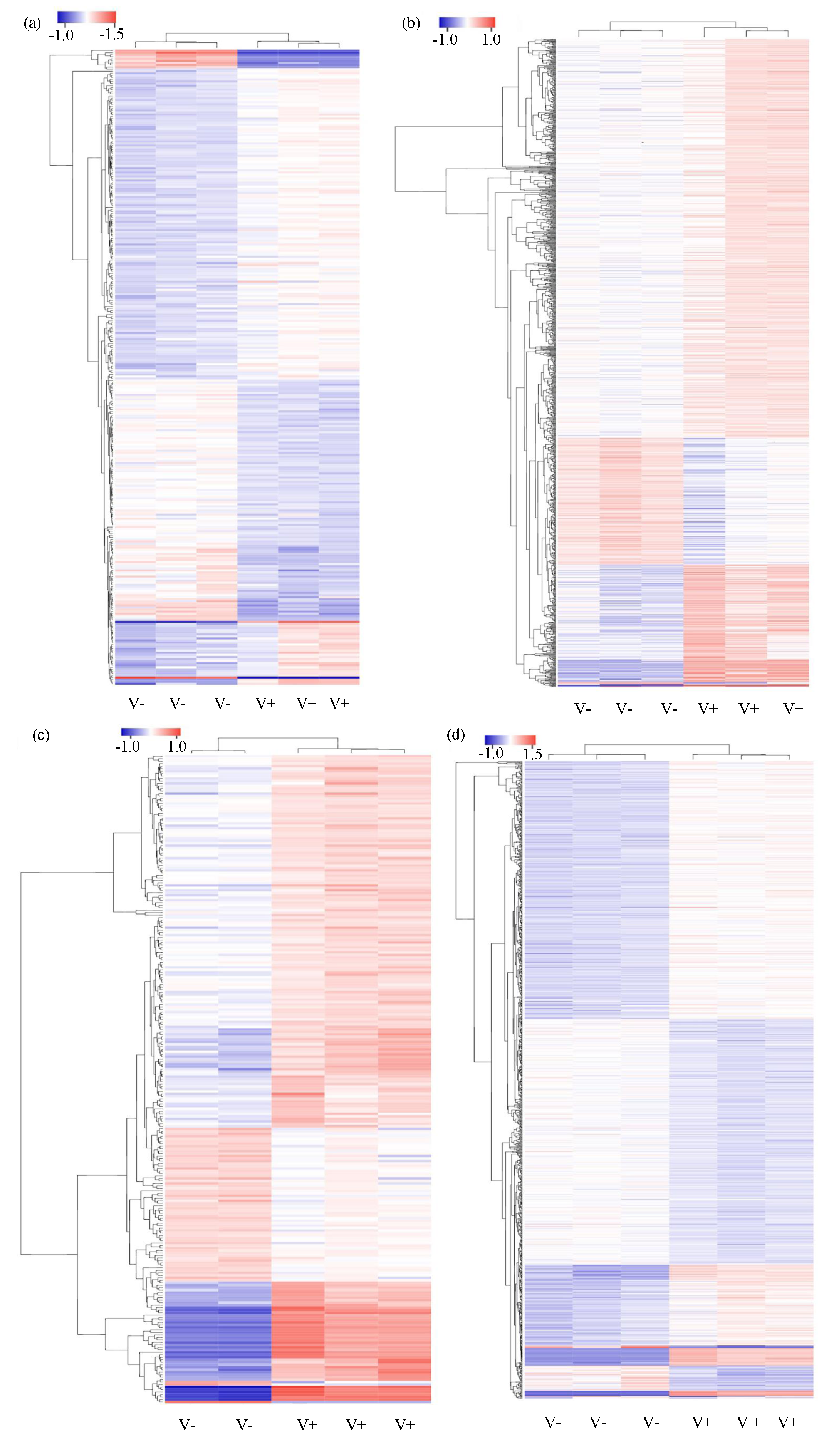

Supplement: S10 Fig — The heatmap shows the dynamic change of DEGs expression in response to partiti-like viruse infection at different stages in S. frugiperda, including larvae (a), pupae (b), male (c) and female (d). The color key representing the log10 TPM values for each unigene. Red indicates a higher gene expression, blue indicates lower expression. “V+” stand for samples infected by the partiti-like viruses, “V-” stand for negative samples. (TIF) [file ppat.1008467.s010.tif]
