# Supplementary material for: Novel partiti-like viruses are conditional mutualistic symbionts in their normal lepidopteran host, African armyworm, but parasitic in a novel host, Fall armyworm
Source: PLoS Pathog. 2020 Jun 22;16(6):e1008467. doi: 10.1371/journal.ppat.1008467 (PMC7332103; doi:10.1371/journal.ppat.1008467)
Supplement: S1 Table — The accession numbers of samples generated by RNA-seq. (DOCX) [file ppat.1008467.s011.docx]

**S1Table** Host information and data output for each pool of caterpillar samples.

| **Species** | **Sample_ID** | **Rename** | **Library** | **Stage** | **Partiti-like viruses** | **Total_Reads** | **Total_Bases** | **Library accession** |
| --- | --- | --- | --- | --- | --- | --- | --- | --- |
| S. exempta | *Spodoptera exempta* | */* | *Spodoptera exempta* | Larvae, pupae, adults | / | 45317012 | 6,797,551,800 | SRR8655972 |
| S. frugiperda | *Spodoptera frugiperda* | */* | *Spodoptera frugiperda* | Larvae, pupae, adults | / | 44150156 | 6,622,523,400 | SRR8655973 |
| S. exempta | SEAF19 | SeF1- | D1 | Female adults | Negative | 52195732 | 7837811416 | SRR8594176 |
| S. exempta | SEAF20 | SeF2- |  |  |  | 56120828 | 8427549517 |  |
| S. exempta | SEAF21 | SeF3- |  |  |  | 58071458 | 8718708253 |  |
| S. exempta | SEAM13 | SeM1- | C1 | Male adults | Negative | 54128618 | 8121616770 | SRR8594174 |
| S. exempta | SEAM14 | SeM2- |  |  |  | 55062672 | 8255777449 |  |
| S. exempta | SEAM15 | SeM3- |  |  |  | 59801452 | 8967832093 |  |
| S. exempta | SEL1 | SeL1- | A1 | Larvae | Negative | 61444862 | 9218295348 | SRR8594178 |
| S. exempta | SEL2 | SeL2- |  |  |  | 49513190 | 7424395191 |  |
| S. exempta | SEL3 | SeL3- |  |  |  | 55586150 | 8338493570 |  |
| S. exempta | 8-Sep | SeP1- | B1 | Pupae | Negative | 49173004 | 7369872703 | SRR8594180 |
| S. exempta | 9-Sep | SeP2- |  |  |  | 56255404 | 8428309273 |  |
| S. exempta | V1V2SEAF22 | SeF1+ | D2 | Female adults | Positive | 58183990 | 8738435672 | SRR8594175 |
| S. exempta | V1V2SEAF23 | SeF2+ |  |  |  | 53619788 | 8050899805 |  |
| S. exempta | V1V2SEAF24 | SeF3+ |  |  |  | 55936350 | 8394699051 |  |
| S. exempta | V1V2SEAM16 | SeM1+ | C2 | Male adults | Positive | 59726944 | 8961911923 | SRR8594173 |
| S. exempta | V1V2SEAM17 | SeM2+ |  |  |  | 51132448 | 7665355542 |  |
| S. exempta | V1V2SEAM18 | SeM3+ |  |  |  | 54891614 | 8233667718 |  |
| S. exempta | V1V2SEL4 | SeL1+ | A2 | Larvae | Positive | 58120948 | 8722500233 | SRR8594177 |
| S. exempta | V1V2SEL5 | SeL2+ |  |  |  | 61555154 | 9230262186 |  |
| S. exempta | V1V2SEL6 | SeL3+ |  |  |  | 50698282 | 7605955856 |  |
| S. exempta | V1V2SEP10 | SeP1+ | B2 | Pupae | Positive | 53404358 | 8011065327 | SRR8594179 |
| S. exempta | V1V2SEP11 | SeP2+ |  |  |  | 52452342 | 7865742529 |  |
| S. exempta | V1V2SEP12 | SeP3+ |  |  |  | 52112708 | 7814678815 |  |
| S. frugiperda | SF2AF13 | SfF1- | D1 | Female adults | Negative | 55971612 | 8399252040 | SRR8655976 |
| S. frugiperda | SF2AF14 | SfF2- |  |  |  | 48202630 | 7231487684 |  |
| S. frugiperda | SF2AF15 | SfF3- |  |  |  | 56771626 | 8515669124 |  |
| S. frugiperda | SF2AM19 | SfM1- | C1 | Male adults | Negative | 54593822 | 8174967154 | SRR8655978 |
| S. frugiperda | SF2AM20 | SfM2- |  |  |  | 58770716 | 8798664797 |  |
| S. frugiperda | SF2L1 | SfM1+ | A1 | Larvae | Negative | 59671814 | 8947647842 | SRR8655982 |
| S. frugiperda | SF2L2 | SfL1- |  |  |  | 52341528 | 7846210871 |  |
| S. frugiperda | SF2L3 | SfL2- |  |  |  | 51253116 | 7678945608 |  |
| S. frugiperda | SF2P7 | SfL3- | B1 | Pupae | Negative | 49160224 | 7373057296 | SRR8655980 |
| S. frugiperda | SF2P8 | SfP1- |  |  |  | 50528464 | 7583123233 |  |
| S. frugiperda | SF2P9 | SfP2- |  |  |  | 52934756 | 7938548520 |  |
| S. frugiperda | V1V2SF2AF16 | SfP3- | D2 | Female adults | Positive | 53759130 | 8061605294 | SRR8655975 |
| S. frugiperda | V1V2SF2AF17 | SfF1+ |  |  |  | 55721648 | 8361931666 |  |
| S. frugiperda | V1V2SF2AF18 | SfF2+ |  |  |  | 61890722 | 9286523577 |  |
| S. frugiperda | V1V2SF2AM21 | SfF3+ | C2 | Male adults | Positive | 56754048 | 8502144420 | SRR8655977 |
| S. frugiperda | V1V2SF2AM23 | SfM2+ |  |  |  | 59180108 | 8866073564 |  |
| S. frugiperda | V1V2SF2AM24 | SfM3+ |  |  |  | 56340798 | 8446736134 |  |
| S. frugiperda | V1V2SF2L4 | SfL1+ | A2 | Larvae | Positive | 53903570 | 8082509344 | SRR8655981 |
| S. frugiperda | V1V2SF2L5 | SfL*2+* |  |  |  | 48633802 | 7294543954 |  |
| S. frugiperda | V1V2SF2L6 | SfL3+ |  |  |  | 52139234 | 7820451671 |  |
| S. frugiperda | V1V2SF2P10 | SfP1+ | B2 | Pupae | Positive | 56646830 | 8492249571 | SRR8655979 |
| S. frugiperda | V1V2SF2P11 | SfP+2 |  |  |  | 53841394 | 8076600340 |  |
| S. frugiperda | V1V2SF2P12 | SfP+3 |  |  |  | 54905842 | 8235390662 |  |
