# Supplementary material for: Novel partiti-like viruses are conditional mutualistic symbionts in their normal lepidopteran host, African armyworm, but parasitic in a novel host, Fall armyworm
Source: PLoS Pathog. 2020 Jun 22;16(6):e1008467. doi: 10.1371/journal.ppat.1008467 (PMC7332103; doi:10.1371/journal.ppat.1008467)
Supplement: S2 Table — (DOCX) [file ppat.1008467.s012.docx]

**S2 Table** Primers used in this study.

| Primer name | Primer sequence (5'-3') | Instruction | PCR program |
| --- | --- | --- | --- |
| SEIVF1 | CTATTGACGACATTCGTGC | Detection of *Spodoptera exempta* insect virus 1 (SEIV1) and construction of plasmid for qPCR generating standard curve | 30 s at 94 ^o^C, 30 s at 55 ^o^C, and 45 s at 72 ^o^C for 40 cycles |
| SEIVR1 | GCATTCCAAACTGTTTTGC |  |  |
| SEIVF2 | CATCTCTTAGAATGTATATTCTGC | Detection of *S. exempta* insect virus 2 (SEIV2) and construction of plasmid for qPCR generating standard curve |  |
| SEIVR2 | GTCTTACGAAAGTGATGAGC |  |  |
| SEIVF3 | CAAAACAATTATTCTTTAGAATGC | Detection of *S. exempta* insect virus 3 (SEIV3) |  |
| SEIVR3 | CGTATCGTTTAATACAGATGTAGC |  |  |
| ActinF | CCAGAGCAAGAGAGGTATC | Detection of DNA and RNA templates used in this study |  |
| ActinF | ATTTCCTTCTGCATCCTGT |  |  |
| SEPVF | GTGACTGGGAAACTAGTTGTC | Detection of *S. exempta* plant-fungal virus-like virus (SEPV) | 30 s at 94 ^o^C, 30 s at 50 ^o^C, and 45 s at 72 ^o^C for 40 cycles |
| SEPVR | TTGTAGACGCTCCTCAGC |  |  |
| Wsp81F | TGGTCCAATAAGTGATGAAGAAAC | Detection of *Wolbachia* in the three *Spodoptera* species | 30 s at 94 ^o^C, 30 s at 55 ^o^C, and 30 s at 72 ^o^C for 40 cycles |
| Wsp691R | AAAAATTAAACGCTACTCCA |  |  |
| SEIV13F1 | CGATAAGACTGTCCCTGCGTGGCTC | Specific primers for 3’RACE of SEIV1 | 30 s at 94 ^o^C, 30 s at 70 ^o^C, and 2 min at 72 ^o^C for 5 cycles; 30 s at 94 ^o^C, 30 s at 6 ^o^C, and 2 min at 72 ^o^C for 5 cycles |
| SEIV13F2 | GACCACCTTGTCCTTGGCGATGATTC |  |  |
| SEIV13F3 | TTGACCAACTCCCTAAAGAACCACCTG |  |  |
| SEIV15R1 | AGTAAGATGTCAAATGCGATGCGGATG | Specific primers for 5’RACE of SEIV1 |  |
| SEIV15R2 | GAGCCACGCAGGGACAGTCTTATCG |  |  |
| SEIV15R3 | TCTAACCTTCTTCTCACCTTCAGGGACG |  |  |
| SEIV23F1 | CACCGAAAGCGTATGTATGTGATGGTAAAG | Specific primers for 3’RACE of SEIV2 |  |
| SEIV23F2 | GCGGACTGTTTCCCAGCGGTTCA |  |  |
| SEIV23F3 | AGGGAAGTTGCTTGGTGCCTGTCGTA |  |  |
| SEIV25R1 | ACATACGCTTTCGGTGTAGCCGTTGTAA | Specific primers for 5’RACE of SEIV2 |  |
| SEIV25R2 | CGTCCTTCTTTGTAGCGTAACCCCACTT |  |  |
| SEIV25R3 | GGGTTGGGCATTTACCTTTCCGAGA |  |  |
| SEIV3F1 | CAAAATTTCAAAACAATTATTCT | Amplification of genomic fragments of *S. exempta* insect virus 3 (SEIV3) | 30 s at 94 ^o^C, 30 s at 55 ^o^C, and 45 s at 72 ^o^C for 40 cycles |
| SEIV3R1 | CATATCATAATGCCACGTATG |  |  |
| SEIV33F1 | GATTGAAGTTATAATGAGACATCTTCTG | Specific primers for 3’RACE of SEIV3 | 30 s at 94 ^o^C, 30 s at 70 ^o^C, and 2 min at 72 ^o^C for 5 cycles; 30 s at 94 ^o^C, 30 s at 6 ^o^C, and 2 min at 72 ^o^C for 5 cycles |
| SEIV33F2 | GGGGCCTATTTCCTAGCGGCTCTATGT |  |  |
| SEIV33F3 | GGCCTTCGAACCCCATACATACCAGA |  |  |
| SEIV35R1 | GCGGTCTTGACACGACGACGTTTCT | Specific primers for 5’RACE of SEIV3 |  |
| SEIV35R2 | GGTCCTGTCGAATTTCTTGAAGTCATAAG |  |  |
| SEIV35R3 | GCAAATAGCTTCTGTAAACCATTCGGG |  |  |
| Oligo(dT) | TTTTTTTTTTTTTTT | Synthesizing first strand cDNA template |  |
| 3CDS | AAGCAGTGGTATCAACGCAGAGTACTTTTTTTTTTTTTTTTTTTTTTTTTTTTTTMN | Synthesizing first strand cDNA template for amplifying the 3’ end |  |
| Q_T_ | CCAGTGAGCAGAGTGACGAGGACTCGAGCTCAAGCTTTTTTTTTTTTTT | Universal primers for amplification of the 5’ ends (5UPM = 2 µM Q_T_ + 10 µM Q_O_) |  |
| Q_O_ | CCAGTGAGCAGAGTGACG |  |  |
| Long | CTAATACGACTCACTATAGGGCAAGCAGTGGTATCAACGCAGAGT | Universal primers for amplification of the 3’ ends (3UPM = 2 µM Long + 10 µM Short) |  |
| Short | CTAATACGACTCACTATAGGGC |  |  |
| SEIV1-F | GGCATTCCTGATACTGAA | qPCR for SEIV1 | 45 cycles of 95°C for 15 s, 60 °C for 34 s |
| SEIV1-R | GAGTCACGATCCAGTTAA |  |  |
| SEIV1-P | (FAM ) CTCTATCGTGCCTGGAAGAAGATCA (TAMRA ) |  |  |
| SEIV2-F | CCGTTAATCGATCACTTTA | qPCR for SEIV2 |  |
| SEIV2-R | CGAACTTCTTGAAATCATAC |  |  |
| SEIV2-P | (FAM ) CGACCTCATCTAACACCACGG (TAMRA) |  |  |
| Actin-F | CCATCTACGAAGGTTACG | qPCR for actin gene in *S. exempta* |  |
| Actin-R | GTGGTGGTGAAAGAGTAA |  |  |
| Actin-P | (FAM) ATCCTCCGTCTGGACTTGGC (TAMRA) |  |  |
| SEGAPDH-F | CTTCCTACAAGGTCATCTC | qPCR for GAPDH gene in *S. exempta* |  |
| SEGAPDH-R | CGAAGTTGTCATGGATAAC |  |  |
| SEGAPDH-P | (FAM) AACTGTCTCGCTCCTCTCGC (TAMRA) |  |  |
| SpexNPVF | TCTTTGGGACGTACATACG | Detection of *S. exempta* nucleopolyhedrovirus (SpexNPV) and construction of plasmid for qPCR generating standard curve | 30 s at 94 ^o^C, 30 s at 55 ^o^C, and 30 s at 72 ^o^C for 40 cycles |
| SpexNPVR | GGGTCCGTTGTAAAGAGG |  |  |
| SfMNPVF | ATGTATACTCGTTACAGCTATAACC | Detection of *S. frugiperda* nucleopolyhedrovirus (SfMNPV) and construction of plasmid for qPCR generating standard curve |  |
| SfMNPVR | TTGTGGTATGGTTTATTAGTACG |  |  |
| SlNPVF | TAATGTATAGTCGCTACAGTGC | Detection of *S. littoralis* nucleopolyhedrovirus (SlNPV) |  |
| SlNPVR | CTCTTCGCAAATTTAGTACG |  |  |
| HaNPVF | AAAAGAAAAGCCAGCAACG | Detection of *Helicoverpa armigera* nucleopolyhedrovirus (HaNPV) |  |
| HaNPVR | ACGATTGAATGTCACCACG |  |  |
| SpexNPV-F | CCCGTGTACGTAGGAAACAACA | qPCR for SpexNPV | 45 cycles of 95°C for 15 s, 60 °C for 34 s |
| SpexNPV-R | CAACCGCCGCCCTTCT |  |  |
| SpexNPV-P | (FAM) CGAGTACCGCATCAGCCTGGCC (TAMRA) |  |  |
| SfMNPV-F | CCCAACAGATGTTTCAGA | qPCR for SfMNPV |  |
| SfMNPV-R | CAGAGTGAAGGTTCATGA |  |  |
| SfMNPV-P | (FAM)CTCCGTTGCGACCCTGACTA(TAMRA) |  |  |
| TRINITY_DN40578_c0_g1-F | TTGTAGGCAGGTTTGGC | Detection of DEGs from larvae of *S.exempta* with qPCR | 45 cycles of 95°C for 15 s, 60 °C for 34 s |
| TRINITY_DN40578_c0_g1-R | AGTATGGCGTAGCAGGA |  |  |
| TRINITY_DN57212_c1_g1-F | CAGTAAGACACGCTCCT |  |  |
| TRINITY_DN57212_c1_g1-R | AACGCTTCCTACCAGAT |  |  |
| TRINITY_DN61175_c0_g1-F | TAGGCAACCTGATGTG |  |  |
| TRINITY_DN61175_c0_g1-R | TGATGACCGACTGAAA |  |  |
| TRINITY_DN48245_c0_g1-F | CAGTTTCGGTCGTCAGGTTC | Detection of DEGs from pupae of *S.exempta* with qPCR |  |
| TRINITY_DN48245_c0_g1-R | TGGTCTATGGGATTACAAGG |  |  |
| TRINITY_DN55831_c0_g2-F | GGTCTTTGGAGGTGTTTGGT |  |  |
| TRINITY_DN55831_c0_g2-R | TTCTTGCCTGGGAGTGATAG |  |  |
| TRINITY_DN49288_c1_g2-F | ATGAATGAAAACGGCAAC |  |  |
| TRINITY_DN49288_c1_g2-R | AAAAACACTAACACAACA |  |  |
| TRINITY_DN56087_c1_g1-F | CAGTGGCGGGAGTCAAAT | Detection of DEGs from males of *S.exempta* with qPCR |  |
| TRINITY_DN56087_c1_g1-R | CCAAGTCCAGGAGAAGGT |  |  |
| TRINITY_DN57024_c2_g1-F | CGACCTCACAGGAAACCG |  |  |
| TRINITY_DN57024_c2_g1-R | TGGACCCTCGCAGCAACA |  |  |
| TRINITY_DN58800_c0_g1-F | CCAGAGGCAACGCTCACAAA |  |  |
| TRINITY_DN58800_c0_g1-R | TCAACGGACTTCCCAATCAC |  |  |
| TRINITY_DN37756_c0_g2-F | GGGTGAGGGACAGAAA | Detection of DEGs from females of *S.exempta* with qPCR |  |
| TRINITY_DN37756_c0_g2-R | GTAGCCTTATCAAAGCAAT |  |  |
| TRINITY_DN60716_c4_g1-F | CCGCCTGAGGAAGTGAC |  |  |
| TRINITY_DN60716_c4_g1-R | TCGTAATCTGTAAGGGTAT |  |  |
| TRINITY_DN55794_c1_g3-F | CTGGTTTGTCGGATGGTA |  |  |
| TRINITY_DN55794_c1_g3-R | TTCGGATTAGGTCGTAGA |  |  |
| TRINITY_DN60415_c0_g1-F | GCAGACATCGCTCAC |  |  |
| TRINITY_DN60415_c0_g1-R | ATCTCGGCGGCTTTA |  |  |
| TRINITY_DN63254_c1_g1-F | ATCACCGTCACACTGC |  |  |
| TRINITY_DN63254_c1_g1-R | GTTTGCGAACCCTACC |  |  |
| TRINITY_DN61344_c0_g1-F | GCCCAGACCCCATACTA |  |  |
| TRINITY_DN61344_c0_g1-R | AGACGGCACCCTCCTCC |  |  |
| TRINITY_DN40578_c0_g1-F | TTGTAGGCAGGTTTGGC |  |  |
| TRINITY_DN40578_c0_g1-R | AGTATGGCGTAGCAGGA |  |  |
| TRINITY_DN57212_c1_g1-F | CAGTAAGACACGCTCCT |  |  |
| TRINITY_DN57212_c1_g1-R | AACGCTTCCTACCAGAT |  |  |
| TRINITY_DN61175_c0_g1-F | TAGGCAACCTGATGTG |  |  |
| TRINITY_DN61175_c0_g1-R | TGATGACCGACTGAAA |  |  |
| SEACTINF | CCGCTGAGAGGGAAATCG | Reference genes for *S. exempta* used in detection of DEGs |  |
| SEACTINR | TACCCAAGAAGGAAGGCT |  |  |
| SEGAPDHF | TTTCCGTGTTCCTGTTGC |  |  |
| SEGAPDHR | CTTGAGGGGTCCTTGGGC |  |  |
| TRINITY_DN60784_c0_g1-F | AGACATAAAAATACCACCGC | Detection of DEGs from larvae of *S.frugiperda* with qPCR |  |
| TRINITY_DN60784_c0_g1-R | CTACCTTTCCCATACTCCCA |  |  |
| TRINITY_DN49825_c0_g1-F | GAACCTTGCCTCTATCT |  |  |
| TRINITY_DN49825_c0_g1-R | TACACCACTCCTCTCGT |  |  |
| TRINITY_DN56911_c0_g1-F | GTTGATGCGGTGGCTATGTA |  |  |
| TRINITY_DN56911_c0_g1-R | TTCTCCTGTGTCTTGGGTGC |  |  |
| TRINITY_DN63618_c0_g1-F | CTCTCAACCTAAAACCAATC | Detection of DEGs from pupae of *S.frugiperda* with qPCR |  |
| TRINITY_DN63618_c0_g1-R | AGGAAATCCAAAGCGTAACC |  |  |
| TRINITY_DN66470_c1_g1-F | GCCGAGGCTGGCTTTT |  |  |
| TRINITY_DN66470_c1_g1-R | CCTTGCGTGTTGTTCC |  |  |
| TRINITY_DN87409_c0_g1-F | CTTGAGTTGATGGTCGCT |  |  |
| TRINITY_DN87409_c0_g1-R | AACCTGATGGCTGGAGAG |  |  |
| TRINITY_DN56753_c0_g1-F | CGACTGGCTGGTGATGG | Detection of DEGs from males of *S.frugiperda* with qPCR |  |
| TRINITY_DN56753_c0_g1-R | TTGGGTGTGGTGTTTGC |  |  |
| TRINITY_DN56783_c0_g1-F | TTCACTAATCGCCCCCCAGC |  |  |
| TRINITY_DN56783_c0_g1-R | CAGTTCCCATCCATTCTACG |  |  |
| TRINITY_DN60781_c0_g1-F | TGTATCAAAGACACGCC | Detection of DEGs from females of *S.frugiperda* with qPCR |  |
| TRINITY_DN60781_c0_g1-R | CTGAAACCCCTACCAAC |  |  |
| TRINITY_DN48519_c0_g1-F | GTGATTCTCGTCGTTGGA |  |  |
| TRINITY_DN48519_c0_g1-R | TGGTGTGTGCCTGTTTGT |  |  |
| TRINITY_DN68201_c17_g2-F | CGACATCACCACAGCC |  |  |
| TRINITY_DN68201_c17_g2-R | TCCTTGAATCCACCGA |  |  |
| TRINITY_DN56334_c0_g1-F | ATGCCATAACCACTACAC |  |  |
| TRINITY_DN56334_c0_g1-R | AACTTCAACATCACACAA |  |  |
| SFACTINF | TGTCTCCCACACCGTCCCCAT | Reference genes for *S. frugiperda* used in detection of DEGs |  |
| SFACTINR | ACGAACGATTTCCCTCTCAGC |  |  |
| SFGAPDHF | GGTATGGCTTTCCGTGTTC |  |  |
| SFGAPDHR | ACCTGTTCCTCGGTGTAGT |  |  |
