# Supplementary material for: Novel partiti-like viruses are conditional mutualistic symbionts in their normal lepidopteran host, African armyworm, but parasitic in a novel host, Fall armyworm
Source: PLoS Pathog. 2020 Jun 22;16(6):e1008467. doi: 10.1371/journal.ppat.1008467 (PMC7332103; doi:10.1371/journal.ppat.1008467)
Supplement: S3 Table — (DOCX) [file ppat.1008467.s013.docx]

**S3 Table** The influence of partiti-like viruses on survival rates of *S. exempta*.

| Index | V+ (%) | V- (%) | χ^2^_1_ | n † | P value |
| --- | --- | --- | --- | --- | --- |
| Larval mortality | 16.16 (± 6.56) | 15.85 (± 4.83) | 0.00 | 8 | 0.98 |
| Pupation rate | 93.80 (± 5.57) | 88.98 (± 6.70) | 5.04 | 8 | 0.0248* |
| Eclosion rate | 75.94 (± 13.73) | 83.14 (± 5.27) | 7.52 | 8 | 0.0061** |

V+ = partiti-like viruses-infected; V- = non-infected individuals. Larval mortality = proportion of larvae dying before pupation; pupation rate = proportion of surviving larvae that successfully pupated; eclosion rate = proportion of pupae that successfully eclosed. † For larval mortality, pupation rate, eclosion rate, n = number of batches (115, 139, 112, 121 larvae for V+ and 100, 121, 146, 134 larvae for V-).
