# Supplementary material for: Novel partiti-like viruses are conditional mutualistic symbionts in their normal lepidopteran host, African armyworm, but parasitic in a novel host, Fall armyworm
Source: PLoS Pathog. 2020 Jun 22;16(6):e1008467. doi: 10.1371/journal.ppat.1008467 (PMC7332103; doi:10.1371/journal.ppat.1008467)
Supplement: S4 Table — (DOCX) [file ppat.1008467.s014.docx]

**S4 Table** The influence of partiti-like viruses on survival rates of *S. frugiperda.*

| Index | V+ (%) | V- (%) | χ^2^_1_ | n † | P value |
| --- | --- | --- | --- | --- | --- |
| Larval mortality | 25.38 (± 9.07) | 28.22 (± 9.61) | 0.44 | 13 | 0.51 |
| Pupation rate | 91.03 (± 5.66) | 94.97 (± 3.04) | 5.67 | 13 | 0.0218* |
| Eclosion rate | 87.35 (± 6.35) | 72.84 (± 8.15) | 8.82 | 7 | 0.016* |

V+ = partiti-like viruses-infected; V- = non-infected individuals. Larval mortality = proportion of larvae dying before pupation; pupation rate = proportion of surviving larvae that successfully pupated; eclosion rate = proportion of pupae that successfully eclosed. † For larval mortality, pupation rate, n = number of batches (80, 71, 72, 93, 95, 86, 80 larvae for V+ and 76, 72, 127, 62, 61, 74 larvae for V-).
