# Supplementary material for: Novel partiti-like viruses are conditional mutualistic symbionts in their normal lepidopteran host, African armyworm, but parasitic in a novel host, Fall armyworm
Source: PLoS Pathog. 2020 Jun 22;16(6):e1008467. doi: 10.1371/journal.ppat.1008467 (PMC7332103; doi:10.1371/journal.ppat.1008467)
Supplement: S5 Table — (DOCX) [file ppat.1008467.s015.docx]

**S5 Table** The description of transcriptome in the two *Spodoptera* species.

| Species | type | transcripts | genes | transcripts_percent | genes_percent |
| --- | --- | --- | --- | --- | --- |
| *S. exempta* | NR | 114331 | 60092 | 0.3758 | 0.2872 |
|  | Swiss-Prot | 75012 | 39291 | 0.2466 | 0.1878 |
|  | Pfam | 72571 | 36515 | 0.2386 | 0.1745 |
|  | COG | 14314 | 6490 | 0.04705 | 0.03102 |
|  | GO | 70035 | 41041 | 0.2302 | 0.1962 |
|  | KEGG | 59065 | 31513 | 0.1942 | 0.1506 |
|  | Total_anno | 116262 | 61474 | 0.3822 | 0.2938 |
|  | Total | 304213 | 209231 | 1 | 1 |
| *S. frugiperda* | NR | 111682 | 54919 | 0.3591 | 0.264 |
|  | Swiss-Prot | 70698 | 34531 | 0.2273 | 0.166 |
|  | Pfam | 70466 | 33308 | 0.2266 | 0.1601 |
|  | COG | 13384 | 6025 | 0.04303 | 0.02896 |
|  | GO | 66534 | 36587 | 0.2139 | 0.1759 |
|  | KEGG | 54837 | 27145 | 0.1763 | 0.1305 |
|  | Total_anno | 113563 | 56221 | 0.3651 | 0.2702 |
|  | Total | 311025 | 208038 | 1 | 1 |
